# Weight loss and mortality in people living with HIV: a systematic review and meta-analysis

**DOI:** 10.1186/s12879-023-08889-3

**Published:** 2024-01-02

**Authors:** Sarah Almeida Cordeiro, Tainá Costa Pereira Lopes, Antonio Luiz Boechat, Roberta Lins Gonçalves

**Affiliations:** Program in Health Sciences (PPGCIS), Faculty of Medicine, Federal University of Amazonas - UFAM, Afonso Pena Street, 1053, Centro, Manaus, Amazonas Brazil

**Keywords:** HIV, Highly active antiretroviral therapy, Weight loss, Hospitalization, Malnutrition, HIV wasting syndrome, Mortality

## Abstract

**Background:**

In the first reported cases of human immunodeficiency virus (HIV) infection, people living with HIV (PLHIV) suffered weight loss, which was an independent predictor of mortality. Highly active antiretroviral therapy (HAART) has changed this scenario for ideal weight, overweight, and even obesity. However, some PLHIV, even on HAART, continue to lose weight. Thus, the guiding question of the study was: do PLHIV hospitalized using HAART with weight loss have higher mortality than hospitalized PLHIV using HAART without weight loss?

**Method:**

A systematic review and meta-analysis of prospective cohort studies published in English, Spanish, or Portuguese, searched in the MedLine, Embase, and LILACS databases from March 2020, until October 2023, reported by MOOSE. We analyzed the methodological quality and risk of bias using the Joanna Briggs Institute Critical Appraisal Tool for Cohort Studies; used the risk ratio (RR) to calculate the probability of hospitalized PLWH who lost weight dying, applied the random effect model and created the funnel plot. We used the inverse variance test estimated by the Mantel-Haenszel method, considering a 95% confidence interval (CI), heterogeneity (I^2^), total effect size (Z), and significance value of *p* < 0.05. We performed a sensitivity analysis with meta-regression and meta-analyses on subgroups to diagnose influence and outliers. The quality of evidence and strength of recommendation were analyzed using the Grading of Recommendations Assessment, Development, and Evaluation system (GRADE).

**Results:**

We included 10 of the 711 studies identified, totaling 1,637 PLHIV. The studies were from South Africa (1), Canada (1), China (1), Brazil (1), Cameroon (1), Ethiopia (1), Thailand (1), Colombia (1), and Tanzania (2), from 1996 to 2017. The average age of the participants was 33.1 years old, and the male was predominant. The leading causes of hospital admission were related to co-infections, and the average hospitalization time was 20.5 days. The prevalence of death in hospitalized PLHIV using HAART who lost weight was 57.5%, with a 1.5 higher risk of dying (RR: 1.50, 95% CI: 1.03, 2.19, *p* = 0.04) than hospitalized PLHIV who did not lose weight.

**Conclusion:**

We concluded, with a very low confidence level, that that weight loss significantly increased the risk of death in hospitalized PLWH using HAART.

**Trial Registration and funding:**

PROSPERO International Prospective Register of Systematic Reviews CRD42020191246 https://www.crd.york.ac.uk/prospero/display_record.php?ID=CRD42020191246.

**Supplementary Information:**

The online version contains supplementary material available at 10.1186/s12879-023-08889-3.

## Background

The prevalence of human immunodeficiency virus (HIV) continues to be a devastating global public health challenge, with millions of people affected and many losing their lives to the disease [1, 2]. According to the most recent and robust data, there are currently an estimated 38 million people living with human immunodeficiency virus (HIV) worldwide [3]. This figure, provided by the Joint United Nations Programme on HIV/AIDS (UNAIDS), demonstrates the alarming persistence of this global health crisis. The statistics on HIV mortality are equally concerning, with approximately 690,000 people dying from HIV-related causes in 2019 alone [4].

Despite the availability of highly active antiretroviral therapy (HAART) that revolutionized the management of HIV infection, transforming it from a fatal illness to a manageable chronic disease in recent decades [5], there is still a significant burden of hospitalization and premature mortality among people living with HIV (PLHIV) [6].

HAART involves combining different classes of antiretroviral medications to suppress the replication of the virus in the body, thereby slowing down disease progression and improving immune function [6]. Consequently, the mortality rates among PLHIV have significantly decreased, and individuals are now living longer, healthier lives [5].

Despite these advancements, weight loss remains a significant concern in the era of HAART. Previously, HIV-related weight loss was largely attributed to a condition known as wasting syndrome [7]. This syndrome was characterized by involuntary weight loss, muscle wasting, and a general decline in physical health [7–9]. While HAART has reduced the occurrence of wasting syndrome, weight loss remains a critical and often overlooked condition [7]. PLHIV often experience weight loss as a significant clinical manifestation, and its impact on mortality is a matter of concern for healthcare providers [7, 8]. Weight loss is associated with a lower CD4 + cell count and an independent predictor of mortality in PLHIV [7, 8]. Furthermore, evidence suggests an increase in hospitalizations among PLHIV due to weight loss [9–11].

Therefore, weight loss remains a relevant issue among PLHIV, even in the HAART era. This is primarily due to various factors, including medication side effects, gastrointestinal complications, metabolic changes, and nutritional deficiencies [9]. These factors can lead to unintended weight loss, compromising the overall well-being and quality of life of individuals living with HIV [8, 9, 11].

Given the persistence of weight loss concerns among PLHIV, further research is needed to better understand the underlying mechanisms and develop effective interventions. Predicting and understanding these outcomes in PLHIV on HAART remains an area of limited research [6].

This study aims to investigate the relationship between HAART use, weight loss, and associated factors to provide valuable insights for healthcare providers and contribute to the improvement of patient care in the era of HAART. The study hypothesizes a significant association between weight loss and mortality in PLHIV in hospitalized HAART. By investigating this relationship, we aim to fill gaps in the existing research and provide valuable insights into the management and care of PLHIV.

## Methods

This systematic review and meta-analysis were conducted to analyze the association between weight loss and all-cause mortality in hospitalized PLWH on HAART as the primary outcome and mortality in non-hospitalized PLWH as the secondary outcome.

Registration was conducted in accordance with the guidelines for Meta-Analyses of Observational Studies in Epidemiology (MOOSE) [12].

The research protocol was registered in PROSPERO (CRD42020191246) and published in the journal Principles and Practice of Clinical Research [13].

### Eligibility criteria

We included prospective cohort studies, published in English, Spanish, or Portuguese between 1996 and 2023, that studied PLHIV, adults, and elderly people using HAART who were hospitalized and that presented data on morbidity, mortality, and weight loss. We excluded studies with patients on antiretroviral therapy before HAART, gray literature, incomplete articles, abstracts, review articles, editorials, books, academic articles, dissertations, theses, proceedings of scientific events, and articles not available online.

### Search strategy and information sources

We carried out the search between March 27, 2020, and October 30, 2023. We searched the MedLine databases via PubMed, Embase and LILACS, using the descriptors and correlates found in the Medical Subject Heading (MeSH). We also used specific and related descriptors from the Embase database (ENTREE Thesaurus) and Health Sciences descriptors (DeCS). The descriptors and correlates were combined with each other, using the Boolean operator “AND” and “OR”, according to the search strategy in MedLine via PubMed, Embase and LILACS (Supplementary Box S1).

**Fig. 1:**
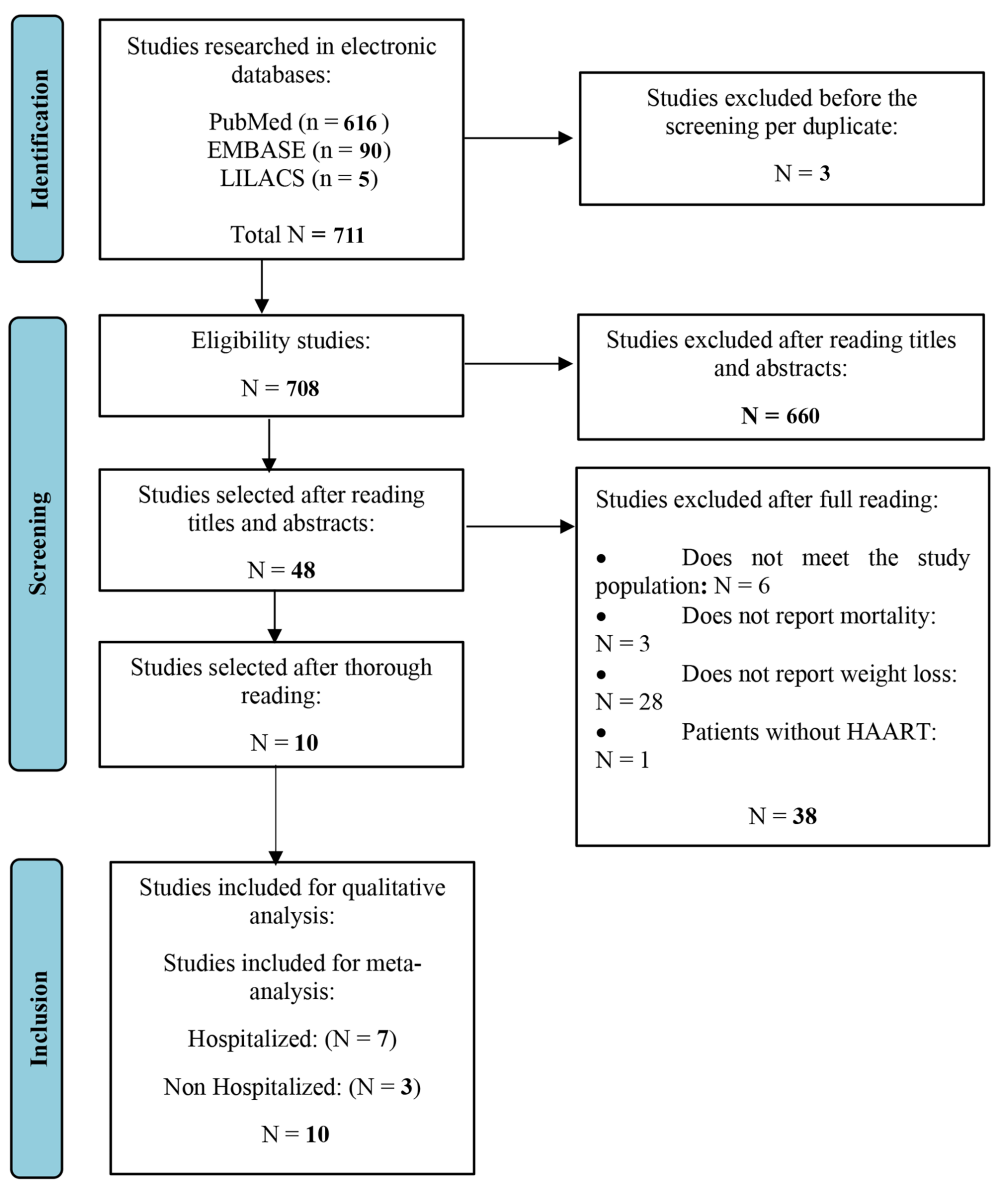
Study selection flowchart for the systematic review and meta-analysis of the effects of weight loss on mortality in PLHIV

When possible, we use the following filters: subject - HIV and/or AIDS; language – English, Portuguese, and Spanish; publication time – between 1996 and 2023; type of studies - only in humans; age group – adults and elderly people; and methodological design – prospective cohort studies. The details of the studies identified in the searches, as well as the demonstration of information from each phase, were prepared according to the flowchart of the Preferred Reporting Items for Systematic Reviews and Meta-Analysis (PRISMA) method [14] (Fig. 1).

### Studies selection and, data extraction

The study selection process was carried out by three reviewers (S.A.C., T.C.P.L., and R.L.G.) independently and blindly, using an online tool from the Rayyan Q.C.R.I. software (Rayyan Q.C.R.I.) from the Qatar Computing Research Institute for Data Analysis [15], which also was used to remove duplicates. Data extraction was carried out by three reviewers (S.A.C., T.C.P.L., and R.L.G.), with discrepancies resolved through group discussion. We extracted the following characteristics from each article: author(s), year, study design, country, length of stay in days, sample size, average age (years), sex, frequency of mortality, and risk of bias for everyone in the study was included in the table (Table 1).

**Table 1:** Main characteristics of included studies

Author(s), year	Study design	Time interval	Country	Risk of bias	Hospitalized / Non-hospitalized	Hospitalization time (days)	Sample Number	Age Average (years old)	Sex F N(%) / M N(%)	Mortality N (%)
Balkema CA *et al*., 2016 [26]	Prospective Observational Cohort	2012–2013	South Africa	Low	Hospitalized	11	26	34.8	18 (69.2) / 8 (30.8)	12 (46.1)
Patterson S *et al*., 2015 [27]	Prospective Cohort	1996–2012	Canada	Moderate	Non-hospitalized	NA	993	41	186 (18.7) / 807 (81.3)	781 (78.6)
Zhao Y *et al*., 2017 [28]	Prospective Observational Cohort	01/2011–12/2011	China	Low	Non-hospitalized	NA	101	38	17 (16.8) / 84 (83.2)	73 (72.2)
Coelho L *et al*., 2016 9 [29]	Observational Cohort	2000–2011	Brazil	Low	Hospitalized	NM	103	35.6	38 (36.8) / 65 (63.2)	64 (62.1)
Chichom MA *et al*., 2015 [30]	Prospective Observational Cohort	2009–2013	Cameroon	Low	Hospitalized	30	9	36	1 (11.1) / 8 (88.9)	4 (44.4)
Fekade D *et al*., 2017 [31]	Prospective Observational Cohort	2009–2013	Ethiopia	Low	Hospitalized	NM	101	33	61 (60.3) / 40 (39.7)	55 (54.4)
Songkhla MN *et al*., 2019 [32]	Multicenter Prospective Cohort	2015–2017	Thailand	Moderate	Hospitalized	NM	41	39	16 (39.0) / 25 (61.0)	35 (85.3)
Caceres DH *et al*., 2016 [33]	Multicenter Prospective Cohort	2008–2011	Colombia	Low	Hospitalized	NM	14	35.2	9 (64.2) 5 (35.8)	12 (85.7)
Sudfeld CR *et al*., 2013 [34]	Prospective Cohort	2006–2009	Tanzania	Low	Hospitalized	NM	195	*	136 (69.7) 59 (30.3)	99 (50.7)
Mugusi SF *et al*., 2012 [35]	Prospective Cohort	2007–2010	Tanzania	Low	Non-hospitalized	NA	54	39.3	31 (57.4) 23 (42.6)	49 (90.7)

Caption: NA: Not applicable; NM: Not mentioned; *The study does not refer to the average age of the participants. Reports by category > 30 years (15.3% of the sample), 30 to 39 years (48.4%), 40 to 50 years (26.7%) and > 50 years (9.7%). Source: the authors (2023)

### Data management

We performed the meta-analysis with Software Review Manager (RevMan) version 5.4.119 [16] (by S.A.C., R.L.G., and A.L.B.). The quality of evidence and strength of recommendation was assessed using the online Guideline Development Tool (GRADEpro G.D.T.) [17] (by S.A.C. and R.L.G.). We used the R software [18] for sensitivity analysis and for managing bibliographic references, the Mendeley Desktop software, version 1.19.822 [19].

### Variables analyzed

We used “weight loss” as the independent variable, defined as a “decrease in existing body weight” [20]. In addition to the independent variable itself, weight loss was also identified as HIV Wasting Syndrome, an AIDS-defining disease characterized by cachexia, weight loss, or weight loss greater than 10% of the patient’s usual weight, equivalent to a stage 3 clinical condition, and 4 AIDS, defined by the World Health Organization (WHO) [20]. Mortality was the dependent variable, identified in the study as the number of deaths or mortality outcomes in PLHIV hospitalized for ≥ 24 h, in days or hours. The hospitalization variable was identified by the concept of the Brazilian Ministry of Health as a “person hospitalized occupying a bed for a period ≥ 24 hours” [21]. HAART results from a combination of antiretroviral medications that, when taken together, can prevent HIV replication by suppressing viral load [22]. In the study, the use of therapy was identified as “yes”: PLHIV using ART or “no”: PLHIV without HAART.

### Bias assessment, quality of evidence and strength of recommendation

Three reviewers (S.A.C., T.C.P.L., and R.L.G.) analyzed the risk of bias of studies selected by the Joanna Briggs Institute (J.B.I.) critical appraisal tool for cohort studies [23]. Disagreements were resolved by consensus. From there, we categorized the studies according to the percentage of positive responses to the questions present in the evaluation instrument. The risk of bias was considered high when the study obtained up to 49% of responses classified as “yes,” moderate when the study obtained 50–69%, and low when the study reached more than 70% as “yes.” Three reviewers (S.A.C., T.C.P.L., and R.L.G.) analyzed the quality of the evidence and the strength of the recommendation was analyzed using the Grading of Recommendations Assessment, Development, and Evaluation (GRADE) system for each outcome, considering the set of evidence through classification into four levels: high, moderate, low, and very low [24]. Disagreements were resolved by consensus.

### Heterogeneity and publication bias

We used Higgins and Thompson’s I² test [25] to evaluate heterogeneity and the random effect model based on it. For sensitivity analysis, we identified outliers and analyzed influence diagnosis, in addition to meta-regression analysis. We created a funnel plot to assess publication bias.

### Statistical analysis

We used the risk ratio (RR) to analyze the probability of hospitalized PLHIV with weight loss resulting in death. We used the inverse variance test (IV) estimated by the Mantel-Haenszel (M-H) method for meta-analysis, considering a 95% confidence interval (95% CI), heterogeneity (I^2^), total effect size (Z) and value of significance of *p* < 0.05 for the average evaluation between the total scores (by S.A.C., R.L.G., and A.L.B.).

## RESULTS

The database search yielded 711 studies, 3 of which were duplicates that were removed. After reading 708 titles and abstracts, we excluded 660 studies, leaving 48 studies that were read in full (supplementary table S2). Of these, we excluded 38 studies for the following reasons: they did not meet the study population (n = 6), did not report mortality (n = 3), did not report weight loss (n = 28), and PLHIV without HAART (n = 1). Therefore, ten full-text studies were accessed and assessed for eligibility based on pre-established criteria and included in the meta-analysis, of which 7 were studies related to mortality in hospitalized PLHIV who lost weight, and three studies were related mortality in PLHIV who lost weight and were not hospitalized (Fig. 1).

### Characteristics of the included studies

We analyzed 1,637 PLHIV, with a mean age of 33.1 years and a predominance of males (n = 1,124, 68.6%). The study period ranged from 1996 to 2017, and the year of publication ranged from 2012 to 2019. The leading causes of hospitalization were related to co-infections, such as tuberculosis, pneumonia, respiratory failure secondary to infections, and sepsis, in addition to weakness neuromuscular and emergency surgeries, such as exploratory laparotomy. The average length of stay was 20.5 days. Five studies (71.4%) did not describe the length of stay.

Of the seven studies included for the primary outcome, which was the association between weight loss and all-cause mortality in PLWH hospitalized on HAART, 489 PLWH hospitalized on ART were analyzed, of which 281 PLWH (57.5%) presented weight loss and death.

In the studies included for the secondary outcome, which was mortality in non-hospitalized PLWH, 1,148 PLWH using HAART were analyzed, of which 903 PLWH (78.7%) had weight loss and died.

Of the ten studies that reported the number of deaths or proportion of deaths in PLHIV with weight loss, the highest mortality rate was 90.7% in the study conducted in Tanzania [34], East Africa, while the lowest mortality rate (44, 4%) was reported in the study carried out in Cameroon, Central Africa [30].

The exposure identified in the included studies was distributed as follows: weight loss found in three (30.0%) of them [32–34]; AIDS-defining illness in two (20.0%) studies [26, 29]; and clinical stages 3 and 4 of the disease in five (50.0%) studies [27, 28, 30, 31, 35]. Other characteristics of the included studies are shown in Table 1.

### Meta-analysis of the effects of weight loss on mortality in hospitalized PLHIV

We performed a meta-analysis with ten prospective cohort studies [26–35]. Seven studies reported weight loss and mortality in hospitalized PLHIV [26, 29–34], three found an association between weight loss and mortality in hospitalized PLHIV [29, 32, 33], and four did not show a significant association between weight loss and mortality in this population [26, 30–32]. The association with mortality was only found in studies from non-African countries.

**Fig. 2:**
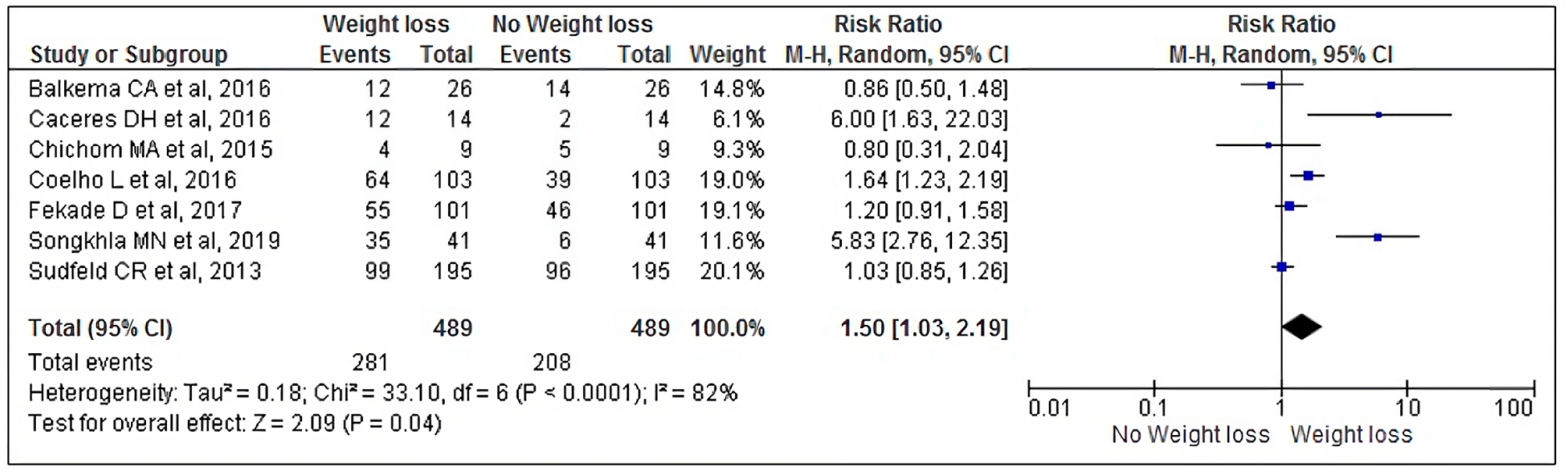
Forest plot of the effects of weight loss on mortality in hospitalized PLHIV

Meta-analysis of seven prospective cohort studies [26, 29–34] involving 489 PLHIV showed that hospitalized PLHIV who lost weight were 1.5 times more likely to die than Hospitalized PLWH who did not lose weight, even when using HAART (RR: 1.50, 95% CI: 1.03, 2.19, *p* = 0.04). The studies included for this outcome showed high heterogeneity (I2 = 82%, *p* < 0.0001). As a result, we constructed a random-effect meta-analysis model to present the data (Fig. 2).

The results can also be visualized in a Drapery plot (Supplementary Figure S1), where the *P* value curves for each estimate from each study are presented, a prediction region indicating heterogeneity and, complementing the forest plot, the other confidence levels (CI 90% and 99%).

We analyzed publication bias using the funnel plot, which demonstrated asymmetry, suggesting the existence of publication bias (Supplementary Figure S2).

### Meta-analysis of the effects of weight loss on non-hospitalized PLHIV mortality

Three prospective cohort studies [27, 28, 35] involving 1,148 PLHIV were included in the meta-analysis for the mortality in non-hospitalized PLHIV.

The meta-analysis result showed that non-hospitalized PLHIV with weight loss had a 3,8-fold greater risk of dying than non-hospitalized PLHIV who did not experience weight loss (RR: 3.84, 95% CI: 2.48, 5.95, *p* = 0.010).

**Fig. 3:**
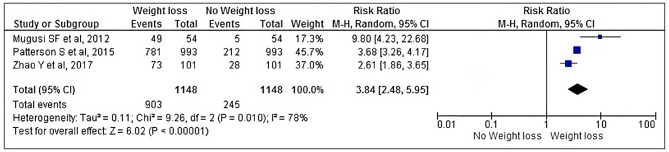
Forest plot of the effects of weight loss on mortality in non-hospitalized PLHIV

We observed high heterogeneity (I^2^ = 78%, *p* < 0.00001), which led us to use a random effect model (Fig. 3).

We assessed publication bias using the funnel plot (Supplementary Figure S3), which demonstrated asymmetry, indicating the existence of publication bias.

The summary of the statistical method, effect estimate, and confidence interval for each outcome are presented in supplementary table S1.

### Sensitivity Analyze and meta-regression analysis

We estimated the variation in heterogeneity between studies by DerSimonian-Laird where tau^2^ = 0.18 and Q test (Chi2) = 33.10, with I^2^ value = 82%, *p* < 0.0001 (95% CI: 63.7-90.9%), in the primary outcome.

When analyzing H = 2.35 (95% CI: 1.66–3.32), it is suggested that the estimated variation in our data may be due to differences in effect size (Supplementary Table S2). In this context, according to the classification proposed by Higgins and Thompson, heterogeneity was characterized as high.

In the presence of high heterogeneity in the included studies, we performed sensitivity analysis with the identification of outliers and diagnosis of influence, in addition to meta-regression analyses and subgroup analysis, which enabled a broad and critical discussion of the results.

The Baujat plot shows the contribution of each study to the overall heterogeneity (Supplementary Figure S4). Our analysis confirmed the notable influence of the study by Songkhla et al., 2019 (Supplementary Figure S5).

**Fig. 4:**
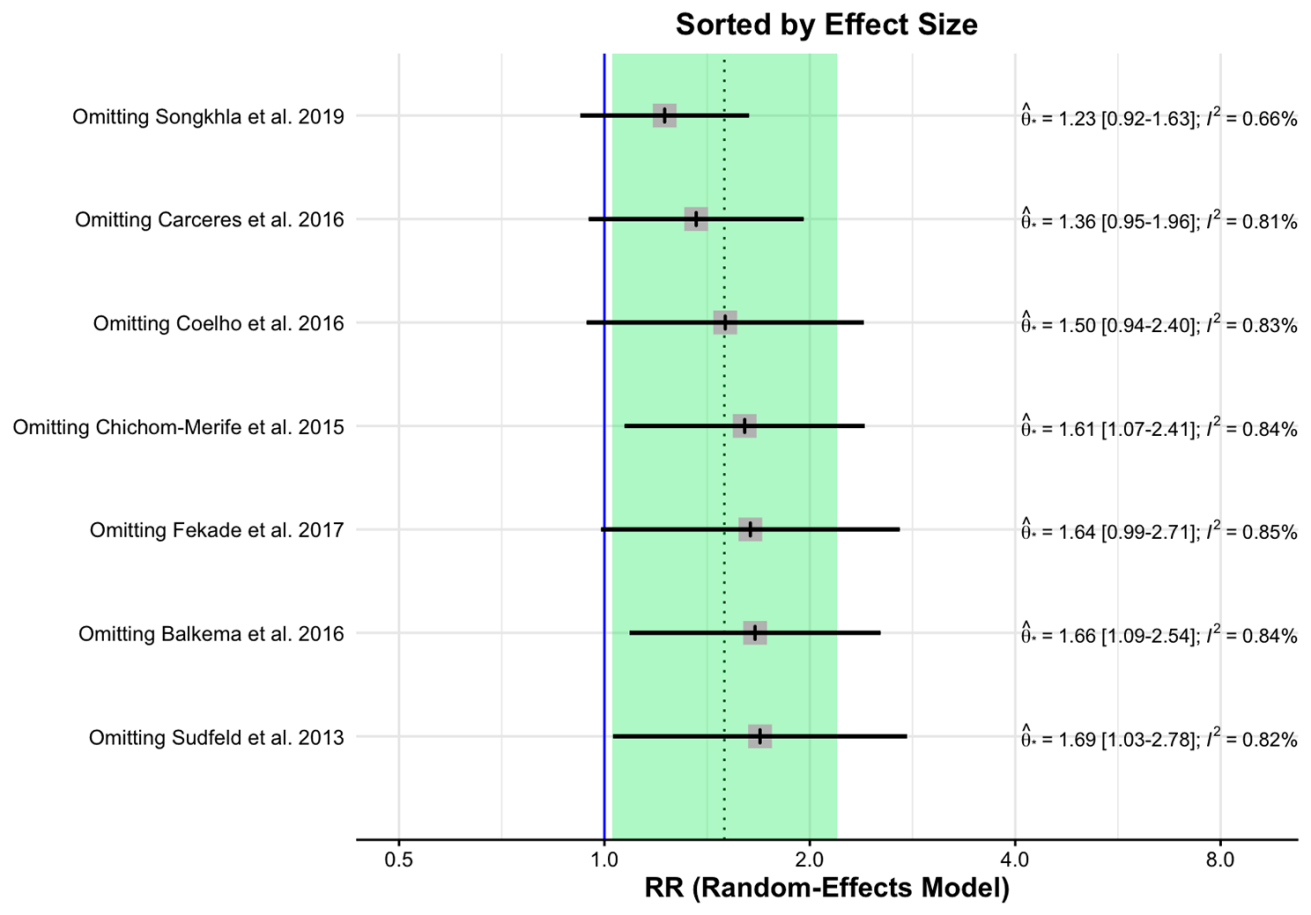
Analysis by the leave-one-out method by effect size between the primary outcome studies

Using the Leave-One-Out method, we performed effect size and heterogeneity (I^2^) analysis of primary outcome studies. It was possible to observe the recalculated effects, with one study omitted at a time. In the graph ordered by effect size (from smallest to largest), you can see the change in the overall effect estimate when different studies are removed. It was possible to observe that the overall effect size is smaller when the study by Songkhla et al., 2019 is removed (Fig. 4).

**Fig. 5:**
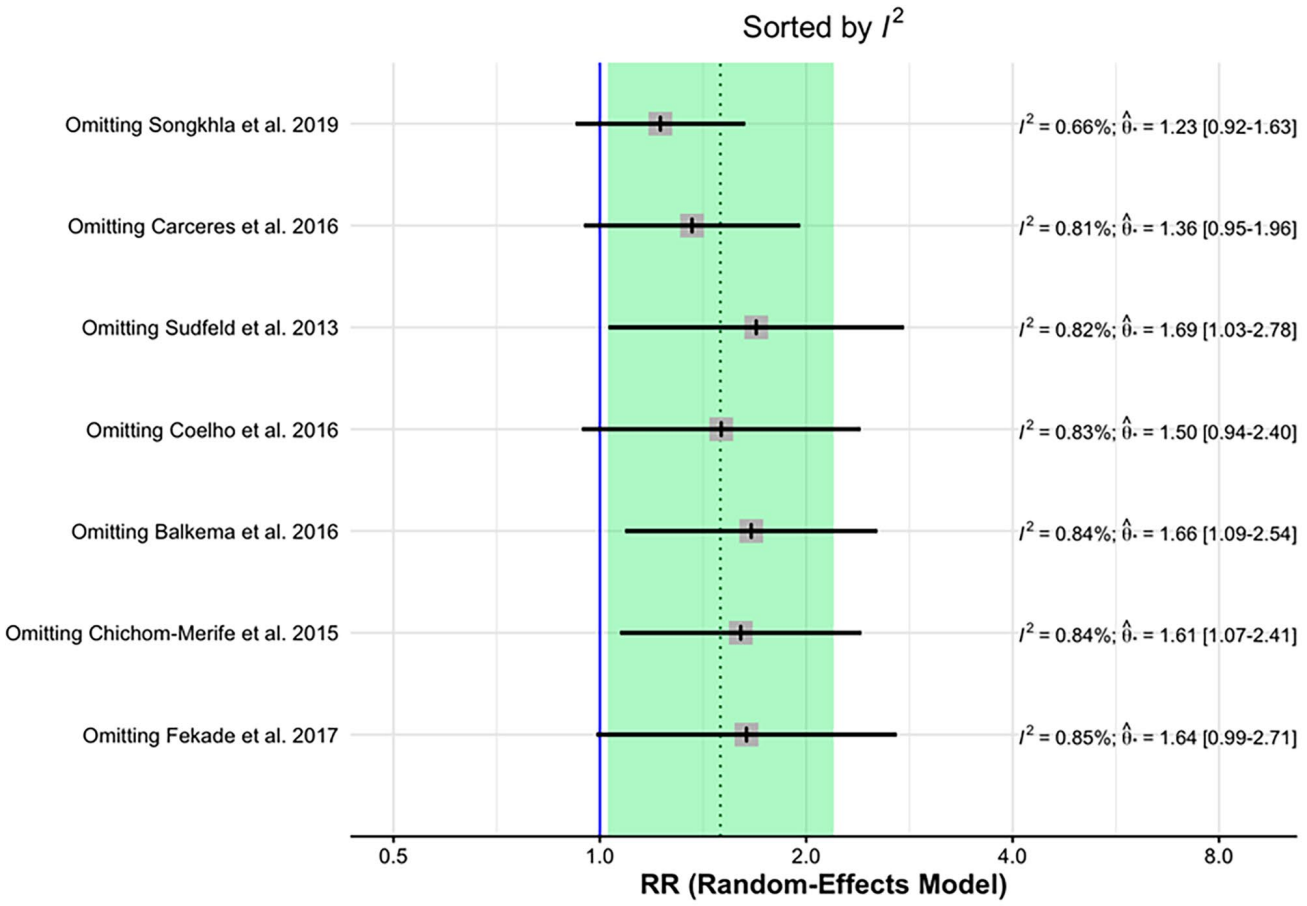
Analysis by the leave-one-out method by I^2^ among the primary outcome studies

Figure 5 presents the heterogeneity graph ordered from lowest to highest, measured by I^2^. This graph shows that the lowest heterogeneity is achieved by omitting the Songkhla et al., 2019 study, confirming the findings that this study contributed to the high heterogeneity in our meta-analysis.

Both the general effect size is smaller, and the heterogeneity decreases from 82 to 66.5%, although, according to the Higgins and Thompson classification, it continues to be high (Table 2).

**Table 2:** Summary of the statistical method, effect estimate and confidence interval by outcome

Outcome	Studies	Participants	Statistical method	Effect estimate
Primary outcome: Mortality in PLHIV with weight loss Hospitalized	7	489	Risk Ratio (M-H, Random, 95% CI)	1.50 (1.03, 2.19)
Secondary outcome: Mortality in PLHIV with weight loss Not hospitalized	3	1148	Risk Ratio (M-H, Random, 95% CI)	3.84 (2.48, 5.95)

Caption: CI: Confidence interval; M-H: Mantel-Haenszel; RR: Risk ratio

For meta-regression, we used the seven included studies to analyze mortality in hospitalized PLHIV. We created a data-adjusted mixed effect model, with tau^2^ estimated by DerSimonian-Laird, to verify whether the gender variable was able to predict mortality in hospitalized PLHIV (Supplementary table S3).

Based on this model, the result of the heterogeneity variance estimate showed: tau^2^ = 0.2111, and I^2^ = 84.09%. The random effect meta-analysis model, the I^2^ was 82%, suggesting that the predictor could not explain a difference in heterogeneity. The R^2^ value (0.00%) reinforces our findings that gender could not predict mortality in hospitalized PLHIV. The test for residual heterogeneity (QQ-test) was performed to test whether heterogeneity, not explained by the predictor, was significant (*p* < 0.0001). The moderator’s test (QM-test), with (*p* = 0.66849), showed that the gender variable did not influence the effect size of the studies (Supplementary table S4).

### Subgroup analysis of the effects of weight loss on mortality in hospitalized and non-hospitalized PLHIV

We performed subgroup meta-analyses based on the geographic location of the countries of the included studies, grouped by continent: Africa, America, and Asia; by sample number, grouped into > 100 participants and < 100 participants; and by the quality of the included studies, grouped into studies with moderate risk of bias and low risk of bias (Supplementary table S5).

There was a difference in the risk of dying from PLHIV in HAART, who lost weight, depending on the continent. In America, RR: 2.90, 95% CI: 1.43, 5.88, *p* = 0.003; in Asia, RR: 3.64, 95% CI: 1.64, 8.04, *p* = 0.001) and in the African continent, RR: 0.42 CI 95%: 0.83, 2.41, *p* = 0.20.

In the subgroup analysis based on the quality of the studies, there was a difference in the RR between the studies classified as moderate (RR: 3.97, 95% CI: 2.84, 5.54, *p* < 0.00001) and low quality (RR: 1.74, 95% CI: 1.15, 2.63, *p* < 0.00001). However, the quality subgroup of studies classified as having a moderate risk of bias showed low heterogeneity (I^2^ = 29%), unlike studies with a Low risk of bias (I^2^ = 88%).

In subgroup analyses based on sample size, no statistical difference was found in the effect of weight loss on mortality in PLHIV on HAART by sample size: less than 100 participants (RR: 2.91, 95% CI: 0.87, 9.69, *p* = 0.08) and greater than 100 participants (RR: 1.81, 95% CI: 0.98, 3.34, *p* = 0.06).

### Analysis of risk of bias and methodological quality

The methodological quality of the included studies was considered high, with a low risk of bias, since 80% of the included studies were classified as having a low risk of bias and 20.0% as moderate risk. There was no study classified as having a high risk of bias.

Six studies (60.0%) did not address loss to follow-up nor strategies to deal with incomplete follow-up of their participants [26, 30, 32–35].

Two (20.0%) studies did not report identifying confounding factors or their strategies to deal with them [27, 32]. Two (20.0%) studies were “unclear” about the similarity of groups when assigning participants to exposed and unexposed groups and, or about the validity and reliability of the exposure measurement [27, 28].

The description and individual classification of the risk of bias and methodological quality of the studies are presented in supplementary table S6.

### Analysis of the quality of evidence and strength of recommendation

We considered the quality of evidence to be very low for both the primary and secondary endpoints, mainly motivated by serious inconsistency, imprecision, and publication bias (Supplementary table S7). Therefore, confidence in the effect estimate was considered very limited, and the results of this review, although relevant, should be considered with caution.

## Discussion

This study expanded knowledge on a relevant topic in relation to a potentially neglected aspect of caring for patients with HIV after HAART, which is the association between weight loss and all-cause mortality. To our knowledge, this was the first meta-analysis with this objective in individuals using HAART.

The global estimate of the cohort studies included in this meta-analysis showed that when PLHIV, even on HAART, lose weight, they are at greater risk of dying than when they do not lose weight, whether they are hospitalized or not. Although confidence in this study’s effect estimate is considered very limited, driven by serious inconsistencies, inaccuracies, and publication bias, the results of this meta-analysis have increased knowledge and scientific evidence on this relevant topic. The results of this review suggest that weight loss continues to be a problem and that there are still important gaps to be addressed on this topic.

It seems clear that it is essential to monitor the weight of PLHIV and implement nutritional strategies for people at nutritional risk. It has been shown that despite the enormous advances in HAART, mortality in PLHIV who experience weight loss is still a persistent concern, especially in hospitalized patients [36]. Estimates from 2016 suggest that between 14 and 38% of PLHIV continue to lose weight [37]. Several conditions are associated with weight loss in PLHIV in HAART, including conditions that affect chewing, swallowing, or gastrointestinal motility; neurological disease that affects food intake or perception of hunger or ability to eat; psychiatric illnesses, food insecurity generated by psychosocial or economic concerns, or anorexia due to medications, malabsorption, infections, or tumors [37].

Updated definitions for weight loss in PLHIV have been developed, although the WHO definition [20] is still the most widely used. This generated variations in the incidence and prevalence of HIV wasting syndrome, with even more alarming data. The update encompasses rapid weight reductions and includes underweight PLHIV with no reference weight available, which the previous definition would have excluded. Additionally, the updated definition requires one of the following criteria with an HIV diagnosis: (1) 10% unintentional weight loss at 12 months, (2) 7.5% unintentional weight loss at six months, or (3) unintentional weight loss of more than five months for three months if other causes or factors of weight loss cannot be ruled out [38]. For PLHIV without documented baseline weight, those who meet criteria for HIV wasting syndrome include 5% loss of body cell mass (BCM) at six months; in men, MCC < 35% of total body weight (BW) and body mass index (BMI) < 27 kg/m^2^; in women: MCC < 23% of TCA and BMI < 27 kg/m^2^; or BMI < 20 kg/m^2^, regardless of MCC in men and women [38]. The most recent definition of HIV weight loss syndrome (ACT loss > 10% since baseline, BMI < 20 kg/m^2^, or unintentional ACT loss > 5% within six months and persisting for more than a year) focuses on the rate of weight loss, as well as the reality that patients present at different stages [39].

Our results demonstrated a mortality rate of 57.5% in hospitalized PLHIV on HAART who experienced weight loss. If we had adopted the updated concepts, the results could have been even worse. However, we decided to be cautious and consider the WHO classification.

Although the Cochrane Database of Systematic Reviews review [40] demonstrated that nutritional interventions have no effect on mortality in PLHIV using HAART, a more recent study strongly recommends that nutritional supplements be maintained for this population, as they can increase body weight, stimulate physical performance and functional recovery, and improve quality of life. The WHO recommends nutritional assessment, consisting of anthropometry, clinical and dietary assessment; nutritional guidance and comprehensive support, essential, according to them, at the time of admission and continuous monitoring of PLHIV with weight loss, at any stage of HIV infection, including during treatment with antiretroviral therapy. The WHO reinforces that dietary supplements may be necessary for malnourished people, especially those experiencing food insecurity, to support nutritional recovery [41]. A gap for additional studies is whether reducing weight loss in PLWH on regular HAART will reduce mortality in these people.

### Limitations of the study

This meta-analysis, while providing valuable insights, is not without its limitations. It is essential to acknowledge these limitations to interpret the results and draw appropriate conclusions accurately.

Firstly, the search strategy employed in this study was restricted to include only studies available in PubMed, Embase, and LILACS databases. Furthermore, the inclusion criteria were limited to studies published in English, Spanish, or Portugues. This approach may have inadvertently excluded relevant studies from other databases or written in different languages. As a result, the findings of this study may not be fully representative of the global population, and caution should be exercised when applying these results to populations outside the selected countries.

Additionally, it is worth noting that this study included data from studies conducted in nine countries. While this provides a certain level of diversity, it may still result in the underrepresentation of specific geographic regions, potentially limiting the generalizability of the results. Therefore, it is crucial to consider the limited scope of the included studies when interpreting the findings.

Furthermore, it is essential to recognize that various confounding factors, such as concomitant illnesses and patients’ clinical status, may influence weight loss’s impact on mortality. These factors were not accounted for in this meta-analysis, potentially introducing bias and limiting the ability to attribute the observed effects solely to weight loss. Consequently, the effect estimate presented in this study should be interpreted with great caution.

Considering these limitations, confidence in the generalizability and accuracy of the effect estimate derived from this study is very limited. It is crucial to exercise caution when applying these findings and consider the broader context and potential confounders that this analysis did not address systematically. Further research incorporating a more comprehensive range of databases, languages, and factors influencing weight loss and mortality is recommended to understand better and validate these results.

## Conclusion

In conclusion, the study conducted in PLHIV undergoing HAART highlights a significant association between weight loss and elevated mortality risk. Even with a very low confidence level, this crucial finding carries substantial implications for numerous vital entities, including implementing agencies, ministries of health, hospitals, and various stakeholders responsible for devising and overseeing programs geared toward curbing mortality rates among PLHIV. To address the complexities surrounding weight loss and its impact on PLHIV mortality, future research endeavors must strive for a comprehensive comprehension of this phenomenon and pursue the establishment of more precise estimations. Such concerted efforts will undoubtedly propel the advancement of strategies to combat the pressing issue of mortality among PLHIV.

### Electronic supplementary material

Below is the link to the electronic supplementary material.

Box S1: Search strategy on MedLine via PubMed, Embase, and LILACS

Box S2: Excluded articles

Fig. S1: Drapery plot of studies on weight loss and mortality in hospitalized PLHIV

Fig. S2: Funnel plot of the effects of weight loss on mortality in hospitalized PLHIV

Fig. S3: Funnel plot of the effects of weight loss on mortality in non-hospitalized PLHIV

Fig. S4: Baujat plot of primary outcome studies

Fig. S5: Influence diagnosis of primary outcome studies

Table S1: Summary of the statistical method, effect estimate and confidence interval by outcome

Table S2: Data for the sensitivity analysis of the primary outcome

Table S3: Effect of the gender variable on mortality in PLHIV

Table S4: Data from the sensitivity analysis by meta regression

Table S5: Summary of statistical method, effect estimate and confidence interval by subgroups

Table S6: Methodological quality and risk of bias assessment

Table S7: Evidence profile and summary of results from the GRADE Working Group

## Data Availability

Full access to study data, responsibility for the study integrity, and issues related to data accuracy to be properly investigated and solved: Sarah Almeida Cordeiro, Tainá Costa Pereira Lopes, Antonio Luiz Boechat, and Roberta Lins Gonçalves.

## Abbreviations

AIDS: Acquired Immunodeficiency syndrome
BMI: Body mass index
CI: Confidence interval
HAART: Highly active antiretroviral treatment
HIV: Human immunodeficiency virus
M-H: Mantel-Haenszel
PLHIV: People living with human immunodeficiency virus
WHO: World health Organization
