# Supplementary material for: Weight loss and mortality in people living with HIV: a systematic review and meta-analysis
Source: BMC Infect Dis. 2024 Jan 2;24:34. doi: 10.1186/s12879-023-08889-3 (PMC10762994; doi:10.1186/s12879-023-08889-3)
Supplement: Supplementary file 1 — Box S1: Search strategy on MedLine via PubMed, Embase, and LILACS [file 12879_2023_8889_MOESM1_ESM.docx]

**Box S1.** Search strategy on MedLine via PubMed, Embase, and LILACS

| PubMed | "HIV"[Mesh] OR (Human Immunodeficiency Virus) OR (Immunodeficiency Virus, Human) OR (Immunodeficiency Viruses, Human) OR (Virus, Human Immunodeficiency) OR (Viruses, Human Immunodeficiency) OR (Human Immunodeficiency Viruses) OR (Human T Cell Lymphotropic Virus Type III) OR (Human T-Cell Lymphotropic Virus Type III) OR (Human T-Cell Leukemia Virus Type III) OR (Human T Cell Leukemia Virus Type III) OR (LAV-HTLV-III) OR (Lymphadenopathy-Associated Virus) OR (Lymphadenopathy Associated Virus) OR (Lymphadenopathy-Associated Viruses) OR (Virus, Lymphadenopathy-Associated) OR (Viruses, Lymphadenopathy-Associated) OR (Human T Lymphotropic Virus Type III) OR (Human T-Lymphotropic Virus Type III) OR (AIDS Virus) OR (AIDS Viruses) OR (Virus, AIDS) OR (Viruses, AIDS) OR (Acquired Immune Deficiency Syndrome Virus) OR (Acquired Immunodeficiency Syndrome Virus) OR (HTLV-III) OR "Antiretroviral Therapy, Highly Active"[Mesh] OR (Highly Active Antiretroviral Therapy) OR HAART OR "Weight Loss"[Mesh] OR (Loss, Weight) OR (Losses, Weight) OR (Weight Losses) OR (Weight Reduction) OR (Reduction, Weight) OR (Reductions, Weight) OR (Weight Reductions) OR "Malnutrition"[Mesh] OR (Malnourishment) OR (Nutritional Deficiency) OR (Undernutrition) OR (Nutritional Deficiencies) OR (Malnourishments) OR "Thinness"[Mesh] OR (Leanness) OR (Underweight) OR "HIV Wasting Syndrome"[Mesh] OR (Slim Disease) OR (Wasting Disease, HIV) OR (Wasting Syndrome, HIV) OR (AIDS Wasting Syndrome) OR (Wasting Syndrome, AIDS) OR (HIV Wasting Disease) AND "Hospitalization"[Mesh] OR Hospitalizations OR "Inpatients"[Mesh] OR Inpatient AND "Body Weight"[Mesh] OR (Body Weights) OR (Weight, Body) OR (Weights, Body) AND "Mortality"[Mesh] OR Mortalities OR (Case Fatality Rate) OR (Case Fatality Rates) OR (Rate, Case Fatality) OR (Rates, Case Fatality) OR (Mortality, Excess) OR (Excess Mortalities) OR (Mortalities, Excess) OR (Excess Mortality) OR (Decline, Mortality) OR (Declines, Mortality) OR (Mortality Declines) OR (Mortality Decline) OR (Mortality Determinants) OR (Determinant, Mortality) OR (Mortality Determinant) OR (Determinants, Mortality) OR (Mortality, Differential) OR (Differential Mortalities) OR (Mortalities, Differential) OR (Differential Mortality) OR (Age-Specific Death Rate) OR (Age-Specific Death Rates) OR (Death Rate, Age-Specific) OR (Death Rates, Age-Specific) OR (Rate, Age-Specific Death) OR (Rates, Age-Specific Death) OR (Age Specific Death Rate) OR (Death Rate) OR (Death Rates) OR (Rate, Death) OR (Rates, Death) OR (Mortality Rate) OR (Mortality Rates) OR (Rate, Mortality) OR (Rates, Mortality) |
| --- | --- |
| EMBASE | (('human immunodeficiency virus'/exp OR (aids AND associated AND lentivirus) OR (aids AND associated AND retrovirus) OR (aids AND associated AND virus) OR (aids AND related AND virus) OR (aids AND virus) OR hiv OR (human AND immuno AND deficiency AND virus) OR (immunodeficiency AND associated AND virus) OR (immunodeficiency AND viruses, AND primate) OR lav OR (lav AND aids) OR (lymphadenopathy AND associated AND retrovirus) OR (lymphadenopathy AND associated AND virus) OR (virus, AND lymphadenopathy AND associated) OR 'highly active antiretroviral therapy'/exp OR (antiretroviral AND therapy, AND highly AND active) OR haart OR 'malnutrition'/exp OR (deficient AND nutrition) OR malnourishment OR (severe AND acute AND malnutrition) OR underfeeding OR undernourishment OR undernutrition OR 'underweight'/exp OR thinness OR (weight AND insufficiency) OR 'wasting syndrome'/exp OR (aids AND associated AND weight AND loss) OR (aids AND wasting AND syndrome) OR (disease, AND wasting) OR (hiv AND wasting AND syndrome) OR (immunodeficiency AND associated AND weight AND loss) OR (slim AND disease) OR (syndrome, AND wasting) OR (wasting AND disease) OR (weight AND loss, AND immunodeficiency AND associated)) AND 'body weight loss'/exp OR (body AND weight AND decrease) OR (body AND weight AND reduction) OR (weight AND decrease) OR (weight AND losing) OR (weight AND loss) OR (weight AND reducing) OR (weight AND reduction) OR (weight AND watching)) AND ('hospitalization'/exp OR (hospital AND stay) OR (short AND stay AND hospitalization) OR 'hospital patient'/exp OR (hospitalised AND patient) OR (hospitalised AND patients) OR (hospitalized AND patient) OR (hospitalized AND patients) OR ('in hospital' AND patient) OR ('in hospital' AND patients) OR 'in patient' OR 'in patients' OR inpatient OR inpatients OR (patient, AND hospital)) AND ('body weight'/exp OR (total AND body AND weight) OR (weight, AND body)) AND ('mortality'/exp OR (excess AND mortality) OR (mortality AND model) OR 'hospital mortality'/exp) |
| LILACS | (tw:(MH:''Desnutrição'' OR MH:''Malnutrition'' OR MH:''Desnutrición'' OR (Subalimentação) OR (Subnutrição) OR MH:C18.654.521$ OR MH:SP6.016.052.058$)) OR (tw:(MH:''Magreza'' OR MH:''Thinness'' OR MH:''Delgadez'' OR Magrez OR MH:C23.888.144.828$ OR MH:E01.370.600.115.100.160.120.828$ OR MH:G07.100.100.160.120.828$)) OR (tw:(MH:''Síndrome de Emaciação por Infecção pelo HIV'' OR MH:''HIV Wasting Syndrome'' OR MH:''Síndrome de Emaciación por VIH'' OR (Doença Consumptiva por Infecção pelo HIV) OR (Doença de Emaciação por Infecção pelo HIV) OR (Síndrome Caquética por Infecção pelo HIV) OR (Síndrome de Emaciação pela AIDS) OR MH:C01.778.640.400.520$ OR MH:C01.925.782.815.616.400.520$ OR MH:C01.925.813.400.520$ OR MH:C18.452.915.520$ OR MH:C18.654.940.520$ OR MH:C20.673.480.520$)) OR (tw:(MH:''Terapia Antirretroviral de Alta Atividade'' OR MH:''Antiretroviral Therapy, Highly Active'' OR MH:''Terapia Antirretroviral Altamente Activa'' OR HAART OR (Terapia Antirretroviral) OR (Terapia de Alta Atividade Antirretroviral) OR MH:E02.319.310.075$)) OR (tw:(MH:''HIV'' OR MH:''HIV'' OR MH:''VIH'' OR (HTLV-III) OR (HTLV-III-LAV) OR (LAV-HTLV-III) OR (Vírus Associado a Linfadenopatia) OR (Vírus Linfotrópico para Células T Humanas Tipo III) OR (Vírus Tipo III T-Linfotrópico Humano) OR (Vírus da AIDS) OR (Vírus da Imunodeficiência Humana) OR (Vírus de Imunodeficiência Humana) OR MH:B04.820.650.589.650.350$)) OR (tw:(mh:"perda de peso"OR mh:"Weight Loss" OR mh:"Perdida de Peso" OR (Emagrecimento) OR (Perda de Massa Corporal) OR (Redução de Peso) OR MH:C23.888.144.243.963$ OR MH:G07.345.249.314.120.200.963$ OR MH:SP6.011.042.048.059$)) AND (tw:(MH:''Hospitalização'' OR MH:''Hospitalization'' OR MH:''Hospitalización'' OR (Comunicação de Internação Hospitalar) OR (Internação Hospitalar) OR (Internação Voluntária) OR MH:E02.760.400$ OR MH:N02.421.585.400$ OR MH:VS3.003.001.002$)) OR (tw:(MH:"Pacientes Internados" OR MH:"Inpatients" OR MH:"Pacientes Internos" OR MH:M01.643.470$)) AND (tw:(MH:"Peso corporal" OR MH:"Body Weight" OR MH:"Peso Corporal" OR MH:C23.888.144$ OR MH:E01.370.600.115.100.160.120$ OR MH:E05.041.124.160.750$ OR MH:G07.100.100.160.120$ OR MH:G07.345.249.314.120$ OR MH:SP6.011.042.048.024$)) AND (tw:(MH:"Mortalidade" OR MH:"Mortality" OR MH:"Mortalidad" OR (Aumento da Mortalidade) OR (Aumento de Mortalidade) OR (Coeficiente de Mortalidade) OR (Declínio da Mortalidade) OR (Determinantes da Mortalidade) OR (Determinantes de Mortalidade) OR (Diminuição da Mortalidade) OR (Estatísticas de Mortalidade) OR (Excesso de Mortalidade) OR (Fatores Determinantes de Mortalidade) OR (Fatores de Mortalidade) OR Letalidade OR (Mortalidade Aumentada) OR (Mortalidade Diferencial) OR (Mortalidade Excessiva) OR (Mortalidade por Faixa Etária) OR (Mortalidade por Idade) OR (Razão de Mortalidade Proporcional) OR (Redução da Mortalidade) OR Sobremortalidade OR (Taxa de Casos Fatais) OR (Taxa de Fatalidade) OR (Taxa de Letalidade) OR (Taxa de Mortalidade) OR (Taxa de Mortalidade por Faixa Etária) OR (Taxa de Mortalidade por Idade) OR (Taxas de Mortalidade Específicas por Idade) OR (Índice de Casos Fatais) OR (Índice de Fatalidade) OR (Índice de Letalidade) OR (Índice de Mortalidade) OR MH:E05.318.308.985.550$ OR MH:N01.224.935.698$ OR MH:N06.850.505.400.975.550$ OR MH:N06.850.520.308.985.550$ OR MH:SP3.076.187.173$ OR MH:SP4.046.452.713$ OR MH:SP4.127.413.629.905$ OR MH:SP5.001.002.028.004$ OR MH:SP5.006.052.168.154$)) |

Source: the authors, 2023
