# Supplementary material for: Weight loss and mortality in people living with HIV: a systematic review and meta-analysis
Source: BMC Infect Dis. 2024 Jan 2;24:34. doi: 10.1186/s12879-023-08889-3 (PMC10762994; doi:10.1186/s12879-023-08889-3)
Supplement: Supplementary file 2 — Box S2: Excluded articles [file 12879_2023_8889_MOESM2_ESM.docx]

**Box S2.** Excluded articles

| **N^o^** | **Author / Year** | **Exclusion justification** |
| --- | --- | --- |
| 1 | Abaalkhail F et al 2017 | It does not comply with PECOT. |
| 2 | Adedimeji A et al 2020 | It does not comply with PECOT. It does not report the weight loss of PLHIV. |
| 3 | Adeoti AO et al 2019 | It does not comply with the study design. It does not report the weight loss of PLHIV. |
| 4 | Ajibola G et al 2018 | It does not comply with the study population. Study with a pediatric population. |
| 5 | Akuzawa Y et al 2019 | It does not comply with PECOT. It does not comply with the study design. Case series study. |
| 6 | Alejos B et al 2016 | It does not comply with PECOT. It does not report the weight loss of PLHIV. |
| 7 | Alencar WK et al 2014 | It does not comply with the study population. Study with a population aged ≥ 13 years. |
| 8 | Alfano G et al 2018 | It does not comply with PECOT. It does not comply with the study design. |
| 9 | Alibhai A et al 2017 | It does not comply with PECOT. |
| 10 | Aliyu MH et al 2015 | It does not comply with PECOT. |
| 11 | Allan B et al 2019 | It does not report the weight loss of PLHIV. |
| 12 | Al-Qadi MO et al 2018 | It does not report the weight loss of PLHIV. |
| 13 | Alvaro-Meca A et al 2016 | It does not comply with the study design. Retrospective cohort study. It does not report the weight loss of PLHIV. |
| 14 | Alvi RM et al 2019 | It does not comply with the study design. It does not report mortality. |
| 15 | Antoniolli L et al 2019 | It does not report the weight loss of PLHIV. |
| 16 | Aoki A et al 2018 | It does not comply with PECOT. |
| 17 | Arantes F et al 2017 | It does not comply with the study design. Patients treated before the establishment of HAART. |
| 18 | Arroyo-Sá Nchez AS & Aguirre-Mejía RY 2020 | It does not comply with PECOT. It does not comply with the study design. Retrospective cohort study. |
| 19 | Arterburn D et al 2015 | It does not comply with PECOT. |
| 20 | Arzoglou V et al 2018 | It does not comply with PECOT. |
| 21 | Asensi-Diez R et al 2019 | It does not comply with the study design. |
| 22 | Asher I et al 2016 | It does not comply with the study design. |
| 23 | Ashraf DC et al 2015 | It does not comply with PECOT. It does not report the weight loss of PLHIV. |
| 24 | Asiimwe SB 2016 | It does not report the use of antiretroviral therapy. |
| 25 | Assi, M et al 2006 | It does not comply with the study design. Literature review study. Patients on treatment before the HAART era. |
| 26 | Aston SJ et al 2019 | After complete reading, it does not meet the PECOT. It does not report weight loss. |
| 27 | Attia A et al 2001 | It does not comply with the study design. |
| 28 | Auer E et al 2019 | It does not comply with PECOT. |
| 29 | Auld AF et al 2016 | It does not comply with the study design. Retrospective cohort study. It does not comply with the study population. Study with a population aged ≥ 15 years. |
| 30 | Aung ZZ et al 2018 | It does not comply with the study design. Retrospective cohort study. It does not comply with the study population. Study with a population aged > 15 years. |
| 31 | Avendaño-Ortiz J et al 2020 | It does not comply with PECOT. |
| 32 | Ayas NT et al 2015 | It does not comply with PECOT. |
| 33 | Aylward RE et al 2019 | It does not comply with PECOT. It does not report the weight loss of PLHIV. |
| 34 | Badali H et al 2015 | It does not comply with the study design. |
| 35 | Badr S et al 2015 | It does not comply with the study design. Case-control study. |
| 36 | Bajunaid K et al 2020 | It does not comply with PECOT. |
| 37 | Balkema CA et al 2014 | It does not report the use of antiretroviral therapy. |
| 38 | Bank IEM et al 2017 | It does not comply with PECOT. It does not comply with the study population. |
| 39 | Baptista SB et al 2016 | It does not comply with PECOT. |
| 40 | Barakat LA et al 2015 | It does not report the weight loss of PLHIV. |
| 41 | Baron RB et al 2020 | It does not comply with PECOT. |
| 42 | Baroncelli S et al 2016 | It does not comply with the study population. Study with a pediatric population. |
| 43 | Bartlett AW et al 2018 | It does not comply with the study population. Study with a pediatric population. |
| 44 | Bélard S et al 2016 | After full reading, the study does not report the mortality of patients with weight loss. |
| 45 | Bellamkonda N et al 2020 | It does not comply with PECOT. |
| 46 | Bellissimo F et al 2016 | It does not comply with PECOT. |
| 47 | Berhanu R et al 2016 | It does not comply with PECOT. |
| 48 | Bernal Morell E et al 2016 | It does not comply with the study design. It does not report mortality. |
| 49 | Beyene T et al 2017 | It does not report the weight loss of PLHIV. |
| 50 | Bhering M et al 2019 | It does not comply with PECOT. |
| 51 | Biccard BM et al 2018 | It does not comply with PECOT. It does not report the weight loss of PLHIV. It does not report the use of antiretroviral therapy. |
| 52 | Bijker R et al 2019 | It does not comply with PECOT. It does not report weight loss. |
| 53 | Bijker R et al 2019 | It does not report the weight loss of PLHIV. |
| 54 | Bishop D et al 2019 | It does not comply with PECOT. |
| 55 | Blanke C et al 2017 | It does not comply with PECOT. It does not comply with the study design. |
| 56 | Blevins M et al 2015 | It does not comply with the study population. Study with a population aged > 15 years. |
| 57 | Boccara F et al 2020 | It does not comply with PECOT. It does not report the mortality of PLHIV. |
| 58 | Bodenner D et al 2007 | It does not comply with the study design. |
| 59 | Boettiger DC et al 2015 | It does not report the weight loss of PLHIV. |
| 60 | Boettiger DC et al 2016 | It does not comply with the study population. Study with a pediatric population. |
| 61 | Boge CLK et al 2019 | It does not comply with the study design. Retrospective cohort study. It does not comply with the study population. Study with a pediatric population. |
| 62 | Boigues BCS et al 2018 | It does not comply with the study design. |
| 63 | Bolondi G et al 2020 | It does not comply with PECOT. |
| 64 | Bongiorni MG et al 2017 | It does not comply with PECOT. |
| 65 | Bonnet M et al 2018 | It does not comply with PECOT. Study with a pediatric population. |
| 66 | Bossini N et al 2014 | It does not report the weight loss of PLHIV. |
| 67 | Bradbury M et al 2019 | It does not comply with PECOT. |
| 68 | Brennan AT et al 2018 | It does not report the mortality of PLHIV. |
| 69 | Brittain EL et al | It does not comply with PECOT. It does not report weight loss. |
| 70 | Bruno S et al 2015 | It does not comply with PECOT. |
| 71 | Buchacz K et al 2015 | It does not report the weight loss of PLHIV. |
| 72 | Bülow E et al 2019 | It does not comply with PECOT. |
| 73 | Buzo BF et al 2020 | It does not comply with PECOT. It does not comply with the study design. |
| 74 | Cabello A et al 2021 | It does not comply with the study design. It does not meet the inclusion criteria regarding the year of publication. |
| 75 | Cabello Úbeda A et al 2016 | It does not comply with the study design. |
| 76 | Cain LE et la 2015 | It does not comply with PECOT. |
| 77 | Calkins KL et al 2020 | It does not report the weight loss of PLHIV. |
| 78 | Campos LC et al 2016 | It does not comply with the study design. |
| 79 | Caniglia EC et al 2017 | It does not comply with PECOT. It does not report the weight loss of PLHIV. |
| 80 | Cantudo-Cuenca MR et al 2014 | It does not comply with PECOT. |
| 81 | Cappello C et al 2020 | It does not comply with PECOT. |
| 82 | Carpentier C et al 2020 | It does not comply with the study design. Retrospective cohort study. |
| 83 | Carrat F et al 2019 | It does not comply with PECOT. |
| 84 | Carvalho FR et al 2017 | It does not comply with PECOT. It does not comply with the study population. |
| 85 | Carvalho PVDC et al 2018 | It does not comply with the study design. |
| 86 | Casado JL et al 2014 | It does not report the weight loss of PLHIV. |
| 87 | Casado JL et al 2015 | It does not comply with PECOT. |
| 88 | Castillo P et al 2017 | It does not comply with PECOT. |
| 89 | Caston JJ et al 2016 | It does not comply with PECOT. |
| 90 | Cattaneo D et al 2020 | It does not report the weight loss of PLHIV. |
| 91 | Cavanaugh PK et al 2016 | It does not comply with PECOT. |
| 92 | cCarty TR et al 2020 | It does not comply with PECOT. |
| 93 | Cescon A et al 2013 | It does not report the weight loss of PLHIV. |
| 94 | Chakravarty J et al 2015 | After the complete reading, it does not comply with the study population. Study with a population aged ≥ 15 years. |
| 95 | Chalamilla G et al 2012 | It does not comply with the study population. Study with a population aged > 15 years. |
| 96 | Chalya PL et al | It does not comply with PECOT. |
| 97 | Chas J et al 2015 | It does not comply with the study design. Retrospective cohort study. |
| 98 | Chawana R et al 2019 | It does not comply with PECOT. Study with a pediatric population. It does not comply with the study design. |
| 99 | Chazova IE et al 2019 | It does not comply with PECOT. |
| 100 | Chen H et al 2019 | It does not comply with PECOT. It does not comply with the study design. |
| 101 | Chen M et al 2016 | It does not report the weight loss of PLHIV. |
| 102 | Chen M et al 2017 | It does not comply with the study design. Retrospective cohort study. |
| 103 | Cheung CP et al 2017 | It does not report the weight loss of PLHIV. It does not comply with the study design. Retrospective cohort study. |
| 104 | Chiliza N et al 2018 | It does not comply with the study design. |
| 105 | Cho J et al 2016 | It does not comply with PECOT. It does not comply with the study population. |
| 106 | Choi J et al 2019 | It does not comply with PECOT. |
| 107 | Christian B et al | It does not comply with PECOT. It does not report weight loss. |
| 108 | Cilloniz C et al 2014 | It does not report the weight loss of PLHIV. |
| 109 | Cillóniz C et al 2017 | It does not comply with the study design. Case-control study. |
| 110 | Cioe PA et al 2015 | It does not comply with PECOT. It does not report the weight loss of PLHIV. |
| 111 | COHERE in EuroCoord Working Group* 2015 | It does not report the weight loss of PLHIV. |
| 112 | Cole SR et al 2017 | It does not comply with PECOT. It does not report the weight loss of PLHIV. |
| 113 | Collett LW et al 2019 | It does not comply with the study design. Case series study. |
| 114 | Cooke WR et al 2018 | It does not comply with PECOT. It does not comply with the study population. |
| 115 | Cooray C et al 2019 | It does not comply with PECOT. |
| 116 | Cornejo-Juárez P et al 2003 | It does not comply with the study design. Case report study. |
| 117 | Cornell M et al 2015 | It does not comply with the study population. Study with a population aged ≥ 16 years. It does not comply with the study design. Retrospective cohort study. |
| 118 | Cortellini A et al 2019 | It does not comply with PECOT. |
| 119 | Coşkun O et al 2017 | It does not comply with PECOT. |
| 120 | Costentin CE et al 2018 | It does not comply with the study population. |
| 121 | Crabtree Ramírez B et al 2017 | It does not comply with the study design. It does not report the weight loss of PLHIV. Patients treated before the establishment of HAART. |
| 122 | Cristelli MP et al 2017 | It does not comply with PECOT. It does not report the weight loss of PLHIV. |
| 123 | Croxford S et al 2017 | After the complete reading, it does not comply with the study population. Study with a population aged ≥ 15 years. |
| 124 | Cunha-Silva M et al 2017 | It does not comply with the study design. |
| 125 | Cutland CL et al 2015 | It does not comply with the study design. Randomized clinical trial study. It does not comply with the study population. Study with a pediatric population. |
| 126 | Czystowska-Kuzmicz M et al 2019 | It does not comply with PECOT. It does not comply with the study population. |
| 127 | da Motta LR et al 2019 | It does not comply with PECOT. |
| 128 | da Silva Escada RO et al 2017 | It does not comply with the study design. Retrospective cohort study. It does not report the weight loss of PLHIV. |
| 129 | Da Silva J et al 2015 | It does not comply with the study design. Ecological study. |
| 130 | da Silva Nascimento J et al 2019 | It does not comply with the study design. Case series study. |
| 131 | Dai L et al 2020 | It does not comply with the study design. |
| 132 | D'Ascenzo F et al 2014 | It does not comply with the study design. |
| 133 | David Morgan M et al 2016 | It does not comply with PECOT. |
| 134 | Davidson B et al 2019 | It does not comply with PECOT. |
| 135 | Davis NL et al 2017 | It does not comply with the study population. Study with a pediatric population. |
| 136 | Davy-Mendez T et al 2019 | It does not comply with PECOT. It does not report the weight loss of PLHIV. |
| 137 | De La Mata NL et al 2016 | It does not comply with the study design. Retrospective cohort study. It does not report the weight loss of PLHIV. |
| 138 | De Lima Mota MA et al 2020 | It does not comply with the study design. |
| 139 | De Smet R et al 2020 | It does not comply with PECOT. |
| 140 | de van der Schueren et al 2018 | It does not comply with PECOT. It does not comply with the study population. |
| 141 | de Vedia L et al 2013 | It does not comply with the study design. It does not report the weight loss of PLHIV. |
| 142 | Delayre T et al 2020 | It does not comply with PECOT. |
| 143 | Della-Torre E et al 2020 | It does not comply with PECOT. |
| 144 | Desai S et al 2016 | It does not comply with the study design. |
| 145 | Di Castelnuovo A et al 2020 | It does not comply with PECOT. It does not comply with the study design. |
| 146 | Di Castelnuovo A et al 2020 | It does not comply with PECOT. |
| 147 | Di Castelnuovo et al 2020 | It does not comply with PECOT. |
| 148 | Di Martino V et al 2015 | It does not report the weight loss of PLHIV. It does not report the use of antiretroviral therapy. |
| 149 | Diaz CM et al 2016 | It does not comply with the study design. Retrospective cohort study. |
| 150 | Dinh TH et al 2018 | It does not comply with the study population. |
| 151 | Dobe I et al 2020 | After complete reading, it does not meet the PECOT. It does not report weight loss. |
| 152 | Drain PK et al 2015 | It does not report the use of antiretroviral therapy. |
| 153 | Dunning J et al 2016 | It does not comply with PECOT. It does not comply with the study design. Randomized clinical trial study. |
| 154 | Durante-Mangoni E et al 2016 | It does not comply with PECOT. |
| 155 | Dwibedi B et al 2014 | It does not comply with PECOT. |
| 156 | Echarte-Morales J et al 2021 | It does not comply with PECOT. It does not meet the inclusion criteria regarding the year of publication. |
| 157 | Edwards R et al 2013 | It does not comply with PECOT. |
| 158 | Ejikunle SD et al 2019 | It does not comply with PECOT. |
| 159 | El-Mallawany NK et al 2016 | It does not comply with the study population. Study with a pediatric population. It does not comply with the study design. |
| 160 | Erlandson KM et al (HAILO) | It does not comply with PECOT. It does not report the weight loss of PLHIV. |
| 161 | Evans RD et al 2017 | It does not report the weight loss of PLHIV. |
| 162 | Eyawo O et al 2017 | It does not report the weight loss of PLHIV. It does not comply with the study design. Retrospective cohort study. |
| 163 | Ezechi LO et al 2016 | It does not comply with PECOT. It does not report the weight loss of PLHIV. |
| 164 | Fabian J et al 2008 | It does not comply with the study design. |
| 165 | Fabris LR et al 2014 | It does not comply with PECOT. It does not comply with the study design. Case series study. |
| 166 | Falasca K et al 2017 | It does not report mortality. It does not comply with PECOT. |
| 167 | Favi E et al 2017 | It does not comply with PECOT. |
| 168 | Fernández Cañabate S & Ortega Valín L 2019 | It does not comply with PECOT. It does not comply with the study design. It does not report the weight loss of PLHIV. |
| 169 | Fernández-Montero JV et al 2014 | It does not comply with the study design. |
| 170 | Fernández-Prada M et al 2018 | It does not comply with the study design. Retrospective cohort study. |
| 171 | Fernández-Ruiz M et al 2020 | It does not comply with PECOT. It does not comply with the study design. |
| 172 | Ferrer E et al 2015 | It does not report the weight loss of PLHIV. |
| 173 | Flancbaum L et al 2005 | It does not comply with PECOT. |
| 174 | Floridia M et al 2017 | It does not comply with the study design. Case series study. |
| 175 | Focaccia R et al 2019 | It does not comply with the study design. It does not report the weight loss of PLHIV. |
| 176 | Fox MP et al 2009 | It does not comply with the study population. |
| 177 | Francini B et al 2018 | It does not report the weight loss of PLHIV. It does not comply with the study design. |
| 178 | Gangcuangco LMA et al 2016 | It does not comply with PECOT. |
| 179 | Gao Y et al 2019 | It does not comply with PECOT. |
| 180 | García-Basteiro AL et al 2016 | It does not comply with the study design. |
| 181 | Garcia-Basteiro AL et al 2019 | It does not comply with PECOT. Study with a pediatric population. |
| 182 | García-Gómez M et al 2015 | It does not comply with PECOT. It does not report the weight loss of PLHIV. |
| 183 | García-Grimshaw M et al 2020 | It does not comply with the study design. Retrospective cohort study. |
| 184 | Gard L et al 2017 | It does not comply with PECOT. It does not comply with the study design. Retrospective cohort study. |
| 185 | Garriga C et al 2015 | It does not comply with PECOT. It does not report the weight loss of PLHIV. |
| 186 | Gathogo E et al 2016 | It does not comply with PECOT. |
| 187 | Ge Z et al 2020 | It does not comply with PECOT. |
| 188 | Geleris J et al 2020 | It does not comply with PECOT. |
| 189 | Geng EH et al (IeDEA) | After full reading, the study does not report the mortality of patients with weight loss. |
| 190 | Gharaibeh L et al 2017 | It does not comply with PECOT. It does not comply with the study population. |
| 191 | Gheorghiță V et al 2019 | It does not comply with the study design. Retrospective cohort study. |
| 192 | Giles ML et al 2016 | It does not comply with PECOT. |
| 193 | Gillis J et al 2016 | It does not report the weight loss of PLHIV. |
| 194 | Giménez E et al 2014 | It does not comply with PECOT. |
| 195 | Glass TR et al 2015 | It does not report the weight loss of PLHIV. |
| 196 | Goehringer F et al | It does not report the weight loss of PLHIV. |
| 197 | Goicoechea M 2020 | It does not comply with PECOT. |
| 198 | González V & Gutiérrez S 2018 | It does not comply with the study population. Study with a pediatric population. |
| 199 | González-García A et al 2017 | It does not comply with the study population. Study with a population aged ≥ 16 years. Patients treated before the establishment of HAART. |
| 200 | González-García A et al 2018 | It does not comply with the study design. Retrospective cohort study. |
| 201 | González-García A et al 2019 | It does not comply with PECOT. It does not comply with the study design. |
| 202 | Gordon KS et al 2020 | It does not comply with PECOT. It does not report the weight loss of PLHIV. |
| 203 | Goswami RP et al 2015 | It does not comply with the study design. It does not report the weight loss of PLHIV. |
| 204 | Goswami RP et al 2017 | It does not comply with the study design. |
| 205 | Grant PM et al 2016 | It does not comply with PECOT. |
| 206 | Greene M et al 2014 | It does not comply with PECOT. It does not report the weight loss of PLHIV. |
| 207 | Greis C et al 2017 | It does not comply with PECOT. |
| 208 | Grilo V & Pereira A 2016 | It does not report the weight loss of PLHIV. |
| 209 | Grint D et al 2015 | It does not report the weight loss of PLHIV. |
| 210 | Grover S et al 2018 | After complete reading, it does not report mortality. |
| 211 | Grover S et al 2020 | It does not report the weight loss of PLHIV. |
| 212 | Guaraldi G et al 2014 | It does not comply with PECOT. It does not report mortality. |
| 213 | Guaraldi G et al 2017 | It does not report mortality. |
| 214 | Guaraldi G et al 2017 | It does not comply with PECOT. It does not comply with the study design. |
| 215 | Guardigni V et al 2016 | It does not comply with the study design. Retrospective cohort study. It does not comply with PECOT. |
| 216 | Guevara-Canales JO et al 2013 | It does not comply with PECOT. |
| 217 | Guillet S et al 2016 | It does not comply with the study design. Case series study. |
| 218 | Guimaraes AO et al 2019 | It does not comply with PECOT. |
| 219 | Guo F et al 2014 | It does not comply with the study design. |
| 220 | Gutierrez-Valencia A et al 2018 | It does not comply with PECOT. |
| 221 | Gwelo AS & Mbishi JV 2019 | It does not comply with the study design. |
| 222 | Haddow LJ et al 2012 | After full reading, the study does not report the mortality of patients with weight loss. |
| 223 | Hall T et al 2020 | It does not comply with PECOT. It does not comply with the study design. Retrospective cohort study. It does not report the weight loss of PLHIV. |
| 224 | Han N et al (AHOD Study e TAHOD Study) 2015 | After complete reading, it does not meet the PECOT. It does not report the weight loss of PLHIV. |
| 225 | Hanna GJ et al 2018 | It does not comply with PECOT. |
| 226 | Hao Y et al 2015 | It does not comply with PECOT. It does not comply with the study population. |
| 227 | Harding R et al 2014 | It does not report mortality. It does not report the weight loss of PLHIV. |
| 228 | Hatleberg CI et al | It does not comply with PECOT. It does not report weight loss. |
| 229 | Hauck C et al 2016 | It does not comply with PECOT. |
| 230 | Hecking M et al 2012 | It does not comply with PECOT. |
| 231 | Heikinheimo T et al 2015 | It does not report the weight loss of PLHIV. Patients treated before the establishment of HAART. |
| 232 | Hentzien M et al 2019 | After complete reading, it does not meet the PECOT. It does not report weight loss. |
| 233 | Henwood BF et al 2015 | It does not comply with PECOT. |
| 234 | Hernández-Cárdenas CM et al 2019 | After complete reading, it does not meet the PECOT. It does not report the weight loss of PLHIV. |
| 235 | Heron JE et al 2019 | It does not report the weight loss of PLHIV. |
| 236 | Herth FJF et al 2020 | It does not comply with PECOT. It does not comply with the study design. Case series study. |
| 237 | Heysell SK et al 2016 | It does not comply with the study population. Study with a population aged ≥ 15 years. |
| 238 | Hiesmayr M et al 2009 | It does not comply with PECOT. |
| 239 | Hill JA et al 2017 | It does not comply with PECOT. |
| 240 | HIV Study writing Group* 2017 | It does not comply with the study population. Study with a population aged > 16 years. |
| 241 | Hodgkinson LM et al 2020 | After complete reading, it does not comply with the study design. It does not comply with the study population. Study with a pediatric population. |
| 242 | Hoenigl M et al 2019 | It does not comply with PECOT. |
| 243 | Hogg RS et al 2017 | It does not comply with PECOT. It does not report the weight loss of PLHIV. It does not comply with the study design. Retrospective cohort study. |
| 244 | Hosseinipour MC et al 2016 | It does not comply with the study design. Randomized clinical trial study. |
| 245 | Howell BA et al 2016 | It does not comply with PECOT. |
| 246 | Hu R et al 2017 | It does not comply with the study design. Retrospective cohort study. |
| 247 | Huang CT et al 2010 | It does not comply with the study design. Patients treated before the establishment of HAART. |
| 248 | Huang P et al 2015 | It does not report the weight loss of PLHIV. |
| 249 | Huang YT et al 2017 | It does not comply with PECOT. |
| 250 | Hue S et al 2020 | It does not comply with PECOT. |
| 251 | Huerga H et al 2019 | After complete reading, it does not comply with the study population. Study with a population aged < 15 years. |
| 252 | Huerga H et al 2020 | It does not comply with the study population. Study with a population aged > 15 years. |
| 253 | Huibers MHW et al 2020 | After complete reading, it does not comply with the PECOT. It does not report the weight loss of PLHIV. |
| 254 | Hulbert A et al 2014 | It does not report the weight loss of PLHIV. |
| 255 | Huntley D et al 2020 | It does not comply with PECOT. |
| 256 | Hurtado JC et al 2019 | It does not comply with PECOT. |
| 257 | Hussain SF et al 2004 | It does not comply with the study population. |
| 258 | Hyle EP et al 2019 | After complete reading, it does not comply with the PECOT. It does not report the weight loss of PLHIV. |
| 259 | Imaizumi Y et al 2020 | It does not comply with PECOT. |
| 260 | Inciarte A et al 2020 | After complete reading, it does not comply with the PECOT. It does not report the weight loss of PLHIV. |
| 261 | Inoue N & Fushimi K 2019 | It does not comply with the study population. |
| 262 | Jabs AW et al 2018 | It does not comply with PECOT. |
| 263 | Jabs DA et al 20015 | It does not comply with PECOT. It does not report the weight loss of PLHIV. |
| 264 | Jabs DA et al 2015 | It does not report the weight loss of PLHIV. |
| 265 | Jaiswal SR et al 2018 | It does not comply with PECOT. |
| 266 | Jaka H et al 2013 | It does not comply with PECOT. It does not comply with the study population. |
| 267 | Janssen S et al 2017 | It does not report the weight loss of PLHIV. |
| 268 | Jantzen C et al 2018 | It does not comply with PECOT. It does not report the weight loss of PLHIV. It does not report the use of antiretroviral therapy. |
| 269 | Jasuja GK et al 2019 | It does not comply with PECOT. |
| 270 | Jeremiah K et al 2014 | It does not comply with PECOT. |
| 271 | Jerene D et al 2006 | After complete reading, it does not comply with the study population. Study with a population aged ≥ 15 years. |
| 272 | Jha A et al 2019 | It does not comply with PECOT. |
| 273 | Jiang H et al 2015 | It does not comply with the study design. |
| 274 | Jiang Y et al 2020 | It does not comply with PECOT. |
| 275 | Jiang YK et al 20158 | It does not comply with the study population. |
| 276 | Jibola G et al 2018 | It does not comply with PECOT. Study with a pediatric population. |
| 277 | Jørgensen CC et al 2016 | It does not comply with PECOT. It does not comply with the study population. |
| 278 | Joseph B et al 2016 | It does not comply with PECOT. |
| 279 | Kalayjian RC et al 2014 | It does not report the weight loss of PLHIV. |
| 280 | Kallas EG et al 2019 | It does not comply with PECOT. |
| 281 | Kamei H et al 2018 | It does not comply with PECOT. It does not comply with the study population. Study with a pediatric population. |
| 282 | Kang R et al 2019 | It does not comply with the study design. Retrospective cohort study. |
| 283 | Kapoor R et al 2015 | It does not comply with PECOT. |
| 284 | Kariv S et al 2018 | It does not comply with PECOT. |
| 285 | Kasereka MC et al 2020 | It does not comply with PECOT. |
| 286 | Kassaye SG et al 2019 | It does not report the weight loss of PLHIV. |
| 287 | Katagira W et al | After full reading, the study does not report the mortality of patients with weight loss. Not all patients were on antiretroviral therapy. |
| 288 | Katz IT et al 2017 | It does not comply with the study design. Retrospective cohort study. |
| 289 | Kawilapat S et al 2019 | It does not comply with the study population. Study with a pediatric population. |
| 290 | Keddy KH et al 2017 | It does not comply with PECOT. It does not comply with the study population. Study with a pediatric population. |
| 291 | Kelly SG et al 2019 | After complete reading, it does not comply with the PECOT. It does not report the weight loss of PLHIV. |
| 292 | Kerber R et al 2019 | It does not comply with the study design. |
| 293 | Kerkhoff AD et al 2020 | It does not comply with PECOT. |
| 294 | Khaitan M et al 2018 | It does not comply with PECOT. It does not comply with the study design. |
| 295 | Khan R et al 2006 | It does not comply with the study population. |
| 296 | Kiertiburanakul S et al 2014 | It does not report the weight loss of PLHIV. |
| 297 | Kim BG et al 2018 | It does not comply with PECOT. It does not comply with the study population. |
| 298 | Kim CY et al 2018 | It does not comply with PECOT. |
| 299 | Kim SU et al 2019 | It does not comply with PECOT. |
| 300 | Kinch A et al 2018 | It does not comply with PECOT. |
| 301 | Kiplagat J et al 2018 | It does not comply with the study population. Study with a population aged ≥ 15 years. It does not comply with the study design. Retrospective cohort study. |
| 302 | Knauf W et al 2018 | It does not comply with PECOT. |
| 303 | Knop V et al 2019 | It does not comply with PECOT. |
| 304 | Knudsen AD et al 2019 | It does not report the weight loss of PLHIV. |
| 305 | Ko RE et al 2019 | It does not comply with the PECOT. It does not comply with the study design. Retrospective study. |
| 306 | Ko Y et al 2014 | It does not comply with the study population. It does not comply with the study design. |
| 307 | Kocayiğit H et al 2021 | It does not comply with PECOT. It does not meet the inclusion criteria regarding the year of publication. |
| 308 | Kociol, Robb D et al 2013 | It does not comply with PECOT. It does not comply with the study design. Randomized clinical trial study. |
| 309 | Koethe JR et al 2010 | It does not comply with the study design. Retrospective cohort study. It does not comply with the study population. Study with a population aged > 15 years. |
| 310 | Komaranchath AS et al 2016 | It does not comply with the study design. Case report study. |
| 311 | Krajinović V et al 2017 | It does not comply with the study design. Case report study. |
| 312 | Kroeber ES 2018 | It does not comply with PECOT. It does not comply with the study design. Retrospective cohort study. |
| 313 | Kruisselbrink R et al 2016 | It does not comply with PECOT. |
| 314 | Ku SW et al 2019 | After complete reading, it does not comply with the PECOT. It does not report the weight loss of PLHIV. |
| 315 | Kumagai S et al 2019 | It does not comply with PECOT. It does not comply with the study population. |
| 316 | Kura S et al 2013 | It does not comply with PECOT. It does not comply with the study design. |
| 317 | Lalueza A et al 2019 | It does not comply with PECOT. It does not comply with the study design. It does not comply with the study population. |
| 318 | Lang J et al 2014 | It does not comply with the study design. Retrospective cohort study. It does not report mortality. |
| 319 | Larsson M et al 2012 | It does not comply with the study design. |
| 320 | Laut KG et al 2019 | It does not comply with PECOT. It does not report the weight loss of PLHIV. |
| 321 | Lee CY et al 2018 | It does not comply with the study design. Retrospective cohort study. |
| 322 | Lee FH et al 2015 | It does not report the weight loss of PLHIV. |
| 323 | Lee JG et al 2015 | It does not comply with PECOT. |
| 324 | Lei C et al 2020 | It does not comply with PECOT. |
| 325 | Leon A et al 2016 | It does not comply with PECOT. |
| 326 | Leone S et al 2019 | It does not comply with PECOT. It does not report mortality. |
| 327 | Lesko CR et al 2016 | It does not report the weight loss of PLHIV. |
| 328 | Lesko CR et al 2020 | It does not comply with PECOT. It does not report the weight loss of PLHIV. |
| 329 | Letendre S et al 2018 | It does not comply with the study design. It does not report the weight loss of PLHIV. |
| 330 | Lewer D et al 2020 | It does not comply with PECOT. |
| 331 | Li C et al 2016 | It does not comply with PECOT. |
| 332 | Li C et al 2020 | It does not comply with PECOT. |
| 333 | Li H et al 2018 | It does not comply with PECOT. |
| 334 | Li H et al 2020 | It does not comply with PECOT. |
| 335 | Li J et al 2018 | It does not comply with PECOT. It does not report the weight loss of PLHIV. |
| 336 | Li L et al 2019 | It does not comply with PECOT. |
| 337 | Lichtner M et al 2015 | It does not comply with PECOT. It does not report the weight loss of PLHIV. |
| 338 | Lin A et al 2019 | It does not comply with PECOT. |
| 339 | Liotta G et al 2013 | It does not comply with the study design. Retrospective cohort study. |
| 340 | Liu C et al 2019 | It does not comply with PECOT. |
| 341 | Liu K et al 2015 | It does not comply with PECOT. |
| 342 | Liu P et al 2017 | It does not report the weight loss of PLHIV. |
| 343 | Liu Q et al 2017 | It does not comply with PECOT. |
| 344 | Liu R et al 2020 | It does not comply with PECOT. |
| 345 | Lodi S et al 2015 | It does not comply with PECOT. It does not report the weight loss of PLHIV. |
| 346 | Lodise TP et al 2020 | It does not comply with PECOT. |
| 347 | Lombo B et al 2015 | It does not comply with PECOT. It does not comply with the study design. |
| 348 | Londoño MC et al 2019 | It does not comply with the study design. It does not comply with the study population. |
| 349 | Lopera MM & Lemos Y 2019 | It does not comply with the study design. It does not report the weight loss of PLHIV. |
| 350 | Lopez A et al 2020 | It does not comply with PECOT. |
| 351 | López-Díaz G et al 2020 | It does not comply with PECOT. |
| 352 | Low DH et al 2019 | It does not comply with PECOT. It does not report the weight loss of PLHIV. |
| 353 | Lu JL et al 2015 | It does not comply with PECOT. |
| 354 | Lu M et al 2016 | It does not comply with PECOT. |
| 355 | Lu M et al 2018 | It does not comply with PECOT. |
| 356 | Lu Y et al 2018 | It does not comply with PECOT. |
| 357 | Luo B et al 2016 | It does not comply with the study design. Retrospective cohort study. It does not report the weight loss of PLHIV. |
| 358 | Luzzati R et al 2019 | It does not comply with PECOT. |
| 359 | Ma X et al 2019 | It does not comply with PECOT. It does not comply with the study design. Retrospective cohort study. |
| 360 | MacLennan CA et al 2017 | It does not comply with the study population. Study with a pediatric population. |
| 361 | Madhi SA et al 2019 | It does not comply with PECOT. Study with a pediatric population. |
| 362 | Mahlab-Guri K et al 2017 | It does not comply with the study design. Retrospective cohort study. It does not report the weight loss of PLHIV. |
| 363 | Makubi A et al 2014 | It does not comply with PECOT. |
| 364 | Makunde WH et al 2012 | It does not comply with PECOT. It does not report the weight loss of PLHIV. |
| 365 | Malagnino V et al 2019 | It does not comply with the study design. Retrospective cohort study. |
| 366 | Malcolm TL et al 2020 | It does not comply with PECOT. |
| 367 | Mallet V et al 2017 | It does not comply with the study design. Retrospective cohort study. |
| 368 | Mallewa J et al 2018 | It does not comply with the study design. Controlled randomized clinical trial study. It does not comply with the study population. Study with a pediatric population. |
| 369 | Marçais A et al 2021 | It does not comply with PECOT. It does not meet the inclusion criteria regarding the year of publication. |
| 370 | Marcy O et al 2018 | Study with a pediatric population. |
| 371 | Martínez-Cuadrón D et al 2018 | It does not comply with PECOT. |
| 372 | Martinez-Perez A et al 2018 | It does not comply with PECOT. |
| 373 | Masri A et al 2014 | It does not comply with PECOT. |
| 374 | Matoga MM et al 2018 | After complete reading, it does not comply with the study design. It does not comply with the study population. Study with a population aged ≥ 15 years. |
| 375 | Mbanya DN et al 2002 | Patients on treatment before the HAART era. |
| 376 | Mbu ET et al 2018 | It does not comply with the study design. |
| 377 | McGinnis GJ et al 2021 | It does not meet the inclusion criteria regarding the year of publication. |
| 378 | McLaren ZM et al 2015 | It does not comply with the study design. |
| 379 | McManus H ey al 2014 | It does not comply with the study design. Case-control study. |
| 380 | McPherson D et al 2018 | It does not report the weight loss of PLHIV. |
| 381 | Medford-Davis LN et al 2016 | It does not comply with PECOT. |
| 382 | Mehta S et al 2009 | It does not comply with the study population. |
| 383 | Meintjes G et al 2015 | It does not report the weight loss of PLHIV. |
| 384 | Mekasha A et al 2020 | It does not comply with the study population. Study with a pediatric population. |
| 385 | Meki CS et al 2018 | It does not comply with PECOT. |
| 386 | Melaku Z et al 2015 | It does not comply with the study design. It does not comply with the study population. Study with a population aged ≥ 15 years. |
| 387 | Menendez ME et al 2015 | It does not comply with PECOT. It does not comply with the study population. |
| 388 | Menéndez, C et al 2008 | It does not comply with PECOT. |
| 389 | Meressa D et al | It does not comply with PECOT. It does not report the weight loss of PLHIV. |
| 390 | Messina FA et al 2014 | It does not report the weight loss of PLHIV. |
| 391 | Meya DB et al 2019 | It does not comply with the study design. Stepped wedge cluster randomized trial study. |
| 392 | Meyers K et al 2015 | It does not comply with the study population. |
| 393 | Miguel-Arias D et al 2016 | It does not comply with PECOT. |
| 394 | Miyano S et al 2013 | It does not comply with PECOT. |
| 395 | Mo P et al 2014 | It does not comply with the study design. It does not report the weight loss of PLHIV. |
| 396 | Mocroft A et al 2014 | It does not comply with the study design. It does not report the weight loss of PLHIV. |
| 397 | Moloney FJ et al 2014 | It does not comply with PECOT. It does not comply with the study population. |
| 398 | Montero F et al 2020 | It does not comply with PECOT. It does not comply with the study population. |
| 399 | Montgomery ND et al 2020 | It does not comply with PECOT. |
| 400 | Montufar Andrade FE et al 2014 | It does not comply with PECOT. It does not report the weight loss of PLHIV. |
| 401 | Moreira J et al 2019 | It does not report the weight loss of PLHIV. It does not report the use of antiretroviral therapy. |
| 402 | Moreira RC et al 2016 | It does not report the weight loss of PLHIV. It does not comply with the study design. Longitudinal cohort study. |
| 403 | Morozova O et al 2013 | It does not comply with PECOT. It does not report the weight loss of PLHIV. |
| 404 | Mu W et al 2014 | It does not comply with the study population. Study with a pediatric population. It does not comply with the study design. Retrospective cohort study. |
| 405 | Muchuweti D & Jönsson KU 2015 | It does not comply with PECOT. |
| 406 | Mueller Y et al 2017 | It does not comply with PECOT. |
| 407 | Mukendi D et al 2017 | It does not comply with the study population. Study with a pediatric population. |
| 408 | Mulenga L et al 2014 | It does not comply with the study population. Study with a population aged > 16 years. |
| 409 | Mulligan, K et al 2005 | It does not comply with the study design. |
| 410 | Mupfumi L et al 2019 | It does not comply with PECOT. It does not comply with the study design. |
| 411 | Mussini C et al 2015 | It does not report the weight loss of PLHIV. |
| 412 | Mutanga JN et al 2019 | It does not comply with the study population. Study with a pediatric population. |
| 413 | Mutembo S et al 2019 | It does not comply with the study design. Retrospective cohort study. |
| 414 | Mutsaerts EAML et al 2019 | It does not comply with the study population. Study with a pediatric population. |
| 415 | Mwita JC et al 2017 | It does not report the weight loss of PLHIV. It does not report the use of antiretroviral therapy. |
| 416 | Myers S et al 2019 | It does not comply with PECOT. Study with a pediatric population. |
| 417 | Myint NPST et al 2018 | It does not comply with the study population. It does not report the use of antiretroviral therapy. |
| 418 | Nadel J et al 2016 | It does not comply with PECOT. It does not comply with the study design. Retrospective cohort study. |
| 419 | Naji P et al 2014 | It does not comply with PECOT. It does not comply with the study population. |
| 420 | Naji P et al 2014 | It does not comply with PECOT. |
| 421 | Nakashima K et al 2018 | It does not comply with the study design. Retrospective cohort study. It does not comply with the study population. |
| 422 | Nayak JJ et al 2013 | It does not comply with the study design. |
| 423 | Ndumbi P et al 2013 | It does not report the weight loss of PLHIV. |
| 424 | Ngo-Giang-Huong N et al 2016 | It does not comply with the study population. Study with a pediatric population. |
| 425 | Nguefack F et al 2016 | It does not comply with the study population. Study with a pediatric population. It does not comply with the study design. |
| 426 | Nguyen CH et al 2018 | It does not comply with PECOT. It does not comply with the study design. |
| 427 | Nirappil FJ et al | It does not comply with PECOT. It does not report the weight loss of PLHIV. |
| 428 | Niyongabo T et al 1999 | Patients treated before the establishment of HAART. It does not comply with the study design. |
| 429 | Norrby M et al 2018 | It does not report the weight loss of PLHIV. |
| 430 | Novelli L et al 2021 | It does not comply with PECOT. It does not meet the inclusion criteria regarding the year of publication. |
| 431 | Ntusi N et al 2016 | It does not comply with PECOT. It does not report the weight loss of PLHIV. |
| 432 | O'Connor G et al 2014 | It does not comply with PECOT. It does not report the weight loss of PLHIV. |
| 433 | Ogata M el al 2017 | It does not comply with PECOT. |
| 434 | Ogbuanu IU et al 2014 | It does not comply with the study population. Study with a pediatric population. |
| 435 | Ogunbayo GO et al 2018 | It does not comply with PECOT. |
| 436 | Okba AM et al 2019 | It does not comply with PECOT. |
| 437 | Oliveira-Sena IV & Werneck GL 2020 | It does not comply with the study design. Case-control study. |
| 438 | Olszewski AJ & Castillo JJ 2016 | It does not comply with PECOT. It does not report the weight loss of PLHIV. It does not report the use of antiretroviral therapy. |
| 439 | Oosten LEM et al 2018 | It does not comply with the study population. Study with a pediatric population. |
| 440 | Oprea C et al 2018 | It does not report the weight loss of PLHIV. |
| 441 | Ordi J et al 2019 | It does not comply with PECOT. It does not comply with the study design. |
| 442 | Ortiz C et al 2014 | It does not comply with PECOT. |
| 443 | Osawa T et al 2020 | It does not comply with the study population. |
| 444 | Pacheco AG et al 2014 | It does not report the weight loss of PLHIV. |
| 445 | Painschab MS et al 2019 | After complete reading, it does not meet the PECOT. It does not report the weight loss of PLHIV. |
| 446 | Palella FJ Jr et al 2016 | It does not report the weight loss of PLHIV. |
| 447 | Palombi L et al 2009 | It does not comply with the study design. It does not comply with the study population. Study with a population aged ≥ 15 years. |
| 448 | Pantazis N et al 2016 | It does not comply with PECOT. It does not report the weight loss of PLHIV. |
| 449 | Pariente A et al 2019 | It does not report the use of antiretroviral therapy. |
| 450 | Parikh RV et al 2014 | It does not comply with PECOT. |
| 451 | Parker A et al et al 2020 | After complete reading, it does not meet the PECOT. |
| 452 | Patel SV et al 2020 | It does not comply with the study design. Retrospective cohort study. |
| 453 | Patsis I et al 2020 | After full reading, the study does not report the mortality of patients with weight loss. |
| 454 | Patterson SB et al 2014 | It does not comply with PECOT. |
| 455 | Pence BW et al 2018 | It does not report the weight loss of PLHIV. |
| 456 | Pereda R et al 2020 | It does not comply with PECOT. |
| 457 | Pereira G et al 2020 | It does not comply with PECOT. |
| 458 | Peri AM et al 2018 | It does not comply with PECOT. |
| 459 | Peter JG et al 2016 | It does not comply with the study design. Controlled randomized clinical trial study. |
| 460 | Peyracchia M et al 2018 | It does not comply with the study design. Case-control study. It does not report the weight loss of PLHIV. |
| 461 | Phakathi B et al 2019 | It does not report the weight loss of PLHIV. |
| 462 | Phan BAP et al 2017 | It does not comply with PECOT. |
| 463 | Phan BAP et al 2020 | It does not comply with PECOT. |
| 464 | Pitter FT et al 2016 | It does not comply with PECOT. |
| 465 | Pizarro-Ortiz M et al 2014 | It does not comply with PECOT. It does not comply with the study population. |
| 466 | Polanco A et al 2014 | After complete reading, it does not meet the PECOT. It does not report the weight loss of PLHIV. |
| 467 | Pongpirul W et al 2018 | It does not report mortality. |
| 468 | Poovorawan K et al 2016 | It does not comply with PECOT. It does not comply with the study design. Retrospective cohort study. |
| 469 | Post FA et al 2014 | It does not report the weight loss of PLHIV. |
| 470 | Postigo A et al 2020 | It does not comply with the study design. |
| 471 | Postorino MC et al (Italian MASTER Cohort) | It does not comply with PECOT. It does not report the weight loss of PLHIV. |
| 472 | Potard V et al 2015 | It does not comply with PECOT. |
| 473 | Poulalhon C et al 2018 | It does not comply with the study population. |
| 474 | Prieto-Tato LM et al 2016 | It does not comply with the study population. Study with a pediatric population. |
| 475 | Punchak M et al 2018 | Study with a pediatric population. |
| 476 | Putot A et al 2015 | It does not comply with the study design. |
| 477 | Qian F et al 2020 | It does not comply with PECOT. |
| 478 | Raffetti E et al 2017 | It does not report the use of antiretroviral therapy. |
| 479 | Raggi P et al 2015 | It does not report the weight loss of PLHIV. |
| 480 | Ragni MV et al 2017 | It does not comply with PECOT. |
| 481 | Ramos JM et al 2020 | It does not comply with PECOT. It does not comply with the study design. |
| 482 | Ramos R et al 2017 | It does not report the use of antiretroviral therapy. |
| 483 | Rasheed AS et al 2019 | It does not comply with PECOT. It does not comply with the study design. |
| 484 | Raubenheimer PJ et al 2019 | It does not report the weight loss of PLHIV. |
| 485 | Ravimohan S et al | It does not comply with PECOT. It does not report the weight loss of PLHIV. |
| 486 | Rawson TM et al 2016 | It does not comply with the study population. |
| 487 | Rebeiro PF et al 2017 | It does not comply with PECOT. |
| 488 | Reddy D & Muckart DJ 2014 | It does not comply with PECOT. It does not comply with the study design. |
| 489 | Redman LA et al 2014 | It does not report the weight loss of PLHIV. |
| 490 | Reniers G et al 2017 | It does not comply with PECOT. |
| 491 | Reniers G et al 2017 | It does not report the weight loss of PLHIV. It does not comply with the study population. Study with a population aged > 15 years. |
| 492 | Rentsch CT et al 2019 | It does not comply with PECOT. |
| 493 | Rider OJ et al 2014 | It does not comply with PECOT. It does not report mortality. |
| 494 | Rivero-Juarez A et al 2017 | It does not comply with PECOT. It does not report the weight loss of PLHIV. |
| 495 | Rockstroh JK et al 2013 | It does not comply with PECOT. |
| 496 | Rodrigues JCL et al 2019 | It does not comply with PECOT. It does not comply with the study population. |
| 497 | Rodríguez-Pérez EG et al 2019 | It does not report the weight loss of PLHIV. It does not report the use of antiretroviral therapy. |
| 498 | Rohr JK et al 2016 | It does not comply with PECOT. |
| 499 | Roland ME et al 2016 | It does not comply with PECOT. |
| 500 | Roland ME et al 2016 | After complete reading, it does not report the weight loss of PLHIV. |
| 501 | Romero-Sánchez MC | It does not comply with the study design. Randomized clinical trial study. |
| 502 | Ronit A et al 2018 | After complete reading, it does not meet the PECOT. It does not report the weight loss of PLHIV. |
| 503 | Rosenberg ES *et al* 2020 | It does not comply with PECOT. It does not comply with the study design. |
| 504 | Rudolf F et al 2017 | It does not comply with PECOT. It does not report the weight loss of PLHIV. |
| 505 | Ryom L et al 2019 | After complete reading, it does not meet the PECOT. It does not report the weight loss of PLHIV. |
| 506 | Sabin CA et al 2013 | It does not report the weight loss of PLHIV. |
| 507 | Sabin CA et al 2017 | Study with a population > 16 years old. It does not report the weight loss of PLHIV. |
| 508 | Sabin CA et al 2018 | It does not comply with PECOT. |
| 509 | Sacks LV & Pendle S 1998 | It does not comply with the study design. Case-control study. |
| 510 | Said EM et al 2020 | It does not comply with PECOT. It does not comply with the study design. Randomized clinical trial study. |
| 511 | Salazar AS et al 2020 | It does not comply with the study design. Retrospective cohort study. |
| 512 | Salvador F et al 2015 | It does not comply with PECOT. It does not comply with the study design. Retrospective cohort study. It does not report the weight loss of PLHIV. |
| 513 | Samji H et al 2015 | It does not comply with PECOT. |
| 514 | Sangil A et al 2015 | It does not comply with PECOT. |
| 515 | Santos CS et al 2020 | It does not comply with PECOT. |
| 516 | Sarfo FS et al 2014 | It does not comply with the study design. |
| 517 | Saylor D et al 2019 | It does not report the use of antiretroviral therapy. |
| 518 | Schmedding M et al 2019 | It does not comply with PECOT. |
| 519 | Schommers P et al 2015 | It does not report the weight loss of PLHIV. |
| 520 | Schommers P et al 2018 | It does not comply with PECOT. It does not report mortality. |
| 521 | Schön T et al 2011 | It does not comply with the study design. Randomized clinical trial study. |
| 522 | Schutz C et al 2019 | After complete reading, it does not meet the PECOT. It does not report the weight loss of PLHIV. |
| 523 | Sellier P et al 2020 | It does not comply with PECOT. It does not report the weight loss of PLHIV. |
| 524 | Serrano-Villar S et al 2014 | It does not comply with the study design. |
| 525 | Seward N et al 2015 | It does not comply with PECOT. |
| 526 | Shah M et al 2013 | It does not comply with PECOT. |
| 527 | Shalaka NS et al 2015 | It does not comply with the study design. |
| 528 | Shamanna SB et al 2016 | It does not comply with the study population. Study with a population aged ≥ 13 years. |
| 529 | Shapiro AE et al 2012 | It does not comply with PECOT. |
| 530 | Sharma G et al 2018 | It does not comply with the study design. Case-control study. |
| 531 | Sharma P et al 2019 | It does not comply with PECOT. |
| 532 | Sharma SK et al 2004 | It does not comply with the study population. Study with a population aged ≥ 13 years. |
| 533 | Sharma SR et al 2017 | It does not comply with the study design. |
| 534 | Shearer K et al 2017 | After complete reading, it does not meet the PECOT. It does not report the weight loss of PLHIV. |
| 535 | Shen T et al 2015 | It does not comply with the study population. Study with a pediatric population. |
| 536 | Shepherd L et al 2014 | It does not comply with the study design. Case-control study. |
| 537 | Shepherd L et al 2016 | It does not comply with PECOT. It does not report the weight loss of PLHIV. |
| 538 | Sherif ZA et al 2020 | It does not comply with PECOT. It does not comply with the study design. |
| 539 | Shi L et al 2019 | It does not comply with PECOT. |
| 540 | Shili-Masmoudi S et al (ANRS CO13 HEPAVIH Study) | After full reading, the study does not report the mortality of patients with weight loss. |
| 541 | Shimazaki T et al 2018 | It does not comply with the study population. |
| 542 | Shimizu K et al 2020 | It does not comply with PECOT. |
| 543 | Shur NF et al 2016 | It does not comply with PECOT. It does not comply with the study design. Retrospective cohort study. |
| 544 | Siedner MJ et al 2019 | It does not comply with PECOT. It does not report the weight loss of PLHIV. |
| 545 | Sili U et al 2018 | It does not comply with the study population. |
| 546 | Silva M et al 2018 | It does not comply with PECOT. It does not report mortality. |
| 547 | Silva TC et al 2017 | It does not comply with the study design. Retrospective cohort study. |
| 548 | Simonsen L et al 2018 | It does not comply with PECOT. |
| 549 | Singh A et al 2016 | It does not comply with the study design. |
| 550 | Singh N et al 2017 | It does not comply with PECOT. |
| 551 | Singh P et al 2015 | It does not comply with the study population. Study with a pediatric population. |
| 552 | Skaar E et al 2021 | It does not comply with PECOT. It does not comply with the study population. It does not meet the inclusion criteria regarding publication time. |
| 553 | Sliwa K et al 2013 | It does not report the use of antiretroviral therapy. |
| 554 | So-Armah KA et al 2016 | It does not report the weight loss of PLHIV. |
| 555 | So-Armah KA et al 2019 | It does not report the use of antiretroviral therapy. |
| 556 | Sohn AH et al 2018 | It does not comply with the study population. Study with a pediatric population. |
| 557 | Solano C et al 2019 | It does not comply with PECOT. |
| 558 | Solano L MF et al 2015 | It does not comply with the study design. Retrospective cohort study. It does not report the weight loss of PLHIV. |
| 559 | Sombogaard F et al 2018 | It does not report the weight loss of PLHIV. It does not comply with the study design. Retrospective cohort study. |
| 560 | Spano JP et al 2019 | It does not comply with PECOT. It does not report the weight loss of PLHIV. |
| 561 | Spradling PR et al 2018 | It does not comply with PECOT. |
| 562 | Sridhar A & Krishna Sagar G 2018 | It does not comply with the study population. Study with a population aged > 14 years. It does not comply with the study design. |
| 563 | Steinhaus N et al 2018 | It does not comply with PECOT. It does not comply with the study population. Study with a population aged > 13 years. |
| 564 | Stone GS et al 2015 | It does not comply with the study design. Retrospective cohort study. It does not report the use of antiretroviral therapy. It does not report the weight loss of PLHIV. |
| 565 | Su S et al 2016 | It does not comply with the study design. Retrospective cohort study. |
| 566 | Subbarao S et al 2015 | It does not report the weight loss of PLHIV. |
| 567 | Sujani Y et al 2018 | It does not comply with the study design. |
| 568 | Sultanova A et al 2013 | It does not comply with PECOT. |
| 569 | Sun B et al 2014 | It does not comply with the study population. It does not report the use of antiretroviral therapy. |
| 570 | Swindells 2018 | It does not comply with the study design. Abstract presented at a scientific event. |
| 571 | Takalkar AA et al 2012 | It does not report mortality. |
| 572 | Takano K et al 2019 | It does not comply with PECOT. |
| 573 | Talbot E et al 2012 | It does not comply with PECOT. |
| 574 | Tamraz B et al 2019 | It does not comply with PECOT. |
| 575 | Tang AM et al 2002 | It does not comply with the study design. |
| 576 | Tang Z et al 2017 | It does not report weight loss in PLHIV. |
| 577 | Tashiro K et al 2019 | It does not comply with the study population. |
| 578 | Taylor BS et al 2017 | It does not comply with PECOT. |
| 579 | Teklu AM & Yirdaw KD, 2016 | It does not comply with the study design. Retrospective cohort study. |
| 580 | Templeton DJ et al 2015 | It does not report the weight loss of PLHIV. |
| 581 | Tepungipame AT et al 2020 | It does not comply with the study design. |
| 582 | Thangaraju S et al 2016 | It does not comply with PECOT. |
| 583 | Thao VP et al 2015 | It does not comply with the study population. Study with a population aged ≥ 15 years. |
| 584 | Thornton AC et al 2017 | It does not report the weight loss of PLHIV. |
| 585 | Ti L et al 2014 | It does not comply with PECOT. |
| 586 | Tiam A et al 2019 | It does not comply with the study population. |
| 587 | Thit SS et al 2017 | After full reading, the study does not report the mortality of patients with weight loss. |
| 588 | Tilley DM et al 2015 | It does not report the weight loss of PLHIV. |
| 589 | Tiniakou E et al 2020 | It does not comply with PECOT. |
| 590 | Tlou B et al 2018 | Study with a pediatric population. |
| 591 | Todd CS et al 2015 | It does not comply with PECOT. |
| 592 | Tokman S et al 2014 | It does not comply with the study design. Case-control study. |
| 593 | Tolsma V et al 2014 | It does not comply with PECOT. |
| 594 | Tominski D et al 2017 | It does not comply with the study design. Retrospective cohort study. |
| 595 | Tomoka T et al 2019 | It does not comply with the study design. |
| 596 | Tornero Patricio S et al 2016 | After complete reading, it does not comply with the PECOT. It does not report mortality. It does not comply with the study design. |
| 597 | Tran DA et al 2014 | It does not comply with PECOT. |
| 598 | Tran T et al 2018 | It does not report mortality. |
| 599 | Tsondai PR et al 2017 | It does not comply with the study design. Retrospective cohort study. |
| 600 | Tsukahara T et al 2017 | It does not comply with the study design. Retrospective cohort study. |
| 601 | Tweya H et al 2017 | It does not comply with the study design. Retrospective cohort study. |
| 602 | Uriarte-Pinto M et al 2018 | It does not comply with PECOT. |
| 603 | Valenzuela-Soltero DM et al 2020 | It does not comply with PECOT. |
| 604 | Vallecillo G et al 2018 | It does not comply with PECOT. It does not report the weight loss of PLHIV. |
| 605 | Vally F et al 2020 | It does not comply with the study design. Retrospective cohort study. |
| 606 | Van der Merwe E et al 2020 | It does not comply with PECOT. It does not report the weight loss of PLHIV. |
| 607 | Van Duin D et al 2018 | It does not comply with PECOT. It does not comply with the study design. |
| 608 | Van Laarhoven A et al 2018 | It does not comply with PECOT. It does not comply with the study population. |
| 609 | Van Laarhoven A et al 2019 | It does not comply with PECOT. |
| 610 | Van Lettow M et al 2015 | It does not comply with the study population. Study with a population aged > 16 years. |
| 611 | Van M et al 2012 | It does not comply with the study population. Study with a population aged ≥ 15 years. |
| 612 | Van Zummeren M et al 2017 | It does not comply with PECOT. |
| 613 | Velásquez-Pérez L & Ramírez-Crescencio MA 2014 | It does not comply with PECOT. |
| 614 | Verdecchia M et al 2018 | It does not comply with the study design. Retrospective cohort study. |
| 615 | Verdugo F et al 2015 | It does not comply with the study design. Retrospective cohort study. |
| 616 | Verhoeff FH et al 2004 | It does not comply with the study population. Study with a pediatric population. |
| 617 | Verna EC et al 2015 | It does not report mortality. |
| 618 | Vernaz N et al 2020 | It does not comply with PECOT. |
| 619 | Viana GMC et al 2017 | It does not comply with the study design. It does not report the use of antiretroviral therapy. |
| 620 | Villamor, E et al 2008 | It does not comply with the study design. Double-blind randomized clinical trial study. |
| 621 | Wakabayashi H et al 2019 | It does not comply with PECOT. It does not comply with the study design. Retrospective cohort study. |
| 622 | Wan YM et al 2018 | It does not comply with PECOT. |
| 623 | Wang B et al 2020 | It does not comply with PECOT. |
| 624 | Wang K et al 2020 | It does not comply with PECOT. It does not comply with the study design. Letter to the editor. |
| 625 | Wang N et al 2020 | It does not comply with PECOT. It does not comply with the study design. |
| 626 | Wang Y et al 2019 | It does not comply with PECOT. |
| 627 | Wang Y et al 2020 | It does not report the use of antiretroviral therapy. |
| 628 | Weber R et al 2015 | It does not comply with the study population. Study with a population aged > 16 years. |
| 629 | Weisberg DF et al 2015 | It does not comply with PECOT. It does not report the weight loss of PLHIV. |
| 630 | Weiser SD et al 2013 | It does not comply with PECOT. It does not report mortality. |
| 631 | Whitworth HS et al 2019 | It does not comply with PECOT. |
| 632 | Wiewel MA et al 2016 | It does not comply with the study design. Case-control study. It does not report the weight loss of PLHIV. |
| 633 | Wójtowicz A et al 2019 | It does not report the weight loss of PLHIV. |
| 634 | Wong GL et al 2015 | It does not comply with the study population. |
| 635 | Wu L et al 2020 | It does not comply with the study design. It does not report the weight loss of PLHIV. |
| 636 | Xiao J et al 2016 | After complete reading, it does not comply with the PECOT. It does not report the weight loss of PLHIV. |
| 637 | Xiao J et al 2015 | It does not comply with the study design. |
| 638 | Xiao J et al 2017 | It does not comply with the study design. Retrospective cohort study. |
| 639 | Xu L et al 2016 | It does not comply with the study design. It does not comply with the study population. |
| 640 | Yagci-Caglayik D et al 2020 | It does not comply with PECOT. It does not comply with the study design. Case series study. |
| 641 | Yang J et al 2018 | It does not comply with the study design. Case-control study. |
| 642 | Youn B et al 2019 | It does not comply with PECOT. It does not comply with the study design. Retrospective cohort study. It does not report the weight loss of PLHIV. |
| 643 | Young JD et al 2014 | It does not comply with PECOT. It does not report the weight loss of PLHIV. |
| 644 | Young N e al 2020 | It does not comply with PECOT. It does not comply with the study design. |
| 645 | Yunda LFI et al 2017 | It does not comply with the study population. Study with a pediatric population. It does not comply with the study design. |
| 646 | Zanuzzi MG et al 2020 | It does not comply with the study design. Observational cross-sectional study. |
| 647 | Zhang F et al 2014 | It does not report the weight loss of PLHIV. It does not comply with the study design. Retrospective cohort study. |
| 648 | Zhang YL et al 2019 | It does not comply with PECOT. |
| 649 | Zhang Z et al 2019 | After complete reading, it does not meet the PECOT. It does not report the weight loss of PLHIV. |
| 650 | Zhao Y et al 2018 | It does not comply with the study design. Retrospective cohort study. |
| 651 | Zhao Y et al 2019 | It does not comply with PECOT. It does not report the weight loss of PLHIV. |
| 652 | Zhao Y et al 2020 | It does not comply with PECOT. |
| 653 | Zheng ZJ et al 2018 | It does not comply with PECOT. |
| 654 | Zhu J et al (NFATP) | After full reading, the study does not report the mortality of patients with weight loss. |
| 655 | Zifodya JS et al 2020 | It does not comply with PECOT. It does not report the weight loss of PLHIV. |
| 656 | Zohar M et al 2015 | It does not comply with the study design. Retrospective cohort study. It does not report the weight loss of PLHIV. |
| 657 | Zoufaly A et al 2014 | It does not report the weight loss of PLHIV. |
| 658 | Ahuja J et al 2023 | It does not report weight loss in PLHIV. |
| 659 | Bannister W et al 2022 | Patients on antiretroviral therapy before HAART. |
| 660 | Muhihi A et al 2022 | Does not meet study design. |
| 661 | Pandey N et al 2022 | It does not report the use of HAART. |
| 662 | Kraef C et al 2021 | It does not comply with PECOT. |
| 663 | Sanhueza-Sanzana C et al 2021 | It does not comply with the study design. Retrospective cohort study. |
| 664 | Palella F et al 2021 | Patients on antiretroviral therapy before HAART. |
| 665 | Wendy P et al 2022 | It does not comply with PECOT. |
| 666 | Moreno et al 2022 | It does not comply with PECOT. |
| 667 | Jessie et al 2021 | It does not report weight loss. |
| 668 | Lee et al 2021 | It does not comply with PECOT. |
| 669 | Rodriguez et al 2021 | It does not comply with PECOT. |
| 670 | Han et al 2021 | It does not report weight loss. |
| 671 | Pérez et al 2021 | It does not comply with the study design. |
| 672 | Milic 2 et al 2021 | Does not comply with PECOT. |
| 673 | Nicol et al 2023 | Does not report weight loss. |
| 674 | Li Wei et al 2021 | Does not comply with PECOT. |
| 675 | Taha et al 2022 | Does not report weight loss. |
| 676 | Pinto et al 2022 | Does not comply with PECOT. |
| 677 | Collins et al 2023 | Does not comply with PECOT. |
| 678 | Buju et al 2022 | Does not report weight loss. |
| 679 | Vergori et al 2021 | Does not comply with PECOT. |
| 680 | Ozturk et al 2021 | Does not meet study design. Does not report weight loss. |
| 681 | Stadelman et al 2021 | Does not comply with PECOT. |
| 682 | Ruden et al 2021 | Does not report mortality in PLHIV. |
| 683 | Solongo et al 2021 | Does not comply with PECOT. |
| 684 | Mweene et al 2021 | Does not comply with PECOT. |
| 685 | MacDuffie et al 2021 | Does not comply with PECOT. |
| 686 | Castelnuovo et al 2021 | Does not comply with the study design. Does not comply with PECOT. |
| 687 | Haruna et al 2021 | Does not report weight loss. Does not comply with PECOT. |
| 688 | Mnguni et al 2023 | Does not comply with PECOT. |
| 689 | Bakewell et al 2022 | Does not comply with PECOT. |
| 690 | Crane et al 2021 | Does not comply with the study design. Does not comply with PECOT. |
| 691 | Telles et al 2021 | Does not report weight loss. |
| 692 | Scott et al 2021 | Does not meet the study population. |
| 693 | Francês et al 2021 | Does not comply with PECOT. |
| 694 | Teles et al 2023 | Does not report weight loss. |
| 695 | Andrade et al 2021 | Does not comply with PECOT. |
| 696 | Zee et al 2023 | Does not meet PECOT. |
| 697 | Castilho et al 2021 | Does not serve the study population. |
| 698 | Correia et al 2021 | Does not meet the study population. |

Caption: PECOT: (P) Population; (E) Exposure; (C): Comparator; (O) Outcome; (T): Time
