# Supplementary material for: Weight loss and mortality in people living with HIV: a systematic review and meta-analysis
Source: BMC Infect Dis. 2024 Jan 2;24:34. doi: 10.1186/s12879-023-08889-3 (PMC10762994; doi:10.1186/s12879-023-08889-3)
Supplement: Supplementary file 3 — Fig. S1: Drapery plot of studies on weight loss and mortality in hospitalized PLHIV [file 12879_2023_8889_MOESM3_ESM.docx]

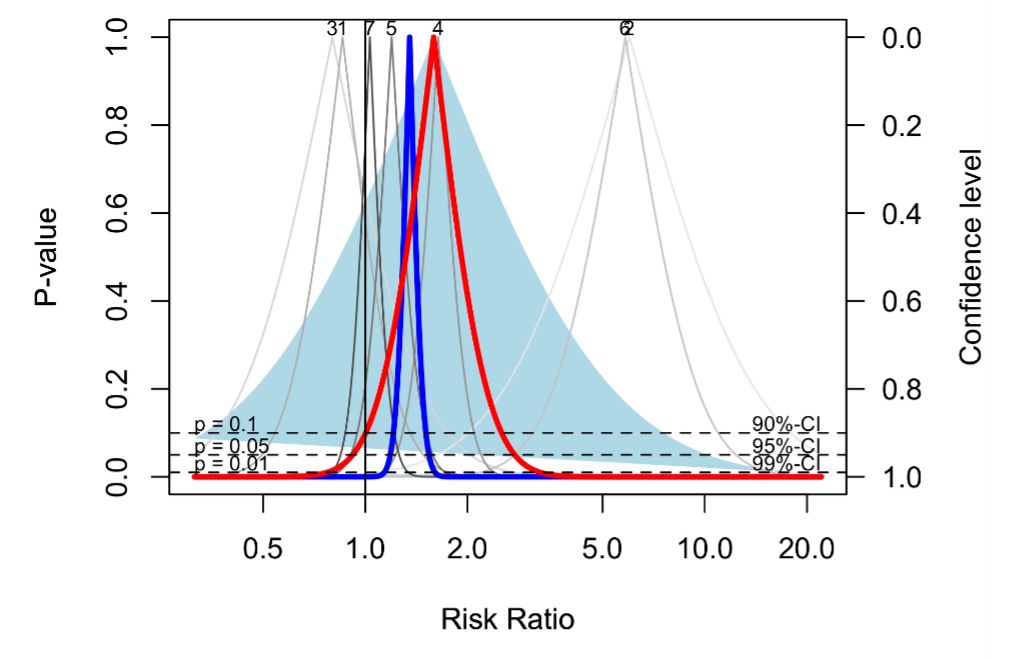


**Fig. S1** Drapery plot of studies on weight loss and mortality in hospitalized PLHIV

(1) Balkema, C. A. et al. (2) Caceres, D. H. et al. (3) Chichom-Mefire, A. et al. (4) Coelho, L. et al. (5) Fekade, D. et al. (6) Songkhla, M. N. et al. (7) Sudfeld, C. R. et al.

In this graph, the P-value curves for each estimate of each individual study are presented, a forecast region indicating heterogeneity (shaded blue), and, complementing the forest graph, the other confidence interval levels (90% and 99% CI) are shown in horizontal dashed lines. For interpretation, the curves in gray correspond to the studies; with the weights corresponding to the random effect model represented in grayscale (studies with higher precision shown in dark gray, studies with low precision in light gray). The red line corresponds to the diamond.
