# Supplementary figures and images for: Weight loss and mortality in people living with HIV: a systematic review and meta-analysis
Source: BMC Infect Dis. 2024 Jan 2;24:34. doi: 10.1186/s12879-023-08889-3 (PMC10762994; doi:10.1186/s12879-023-08889-3)

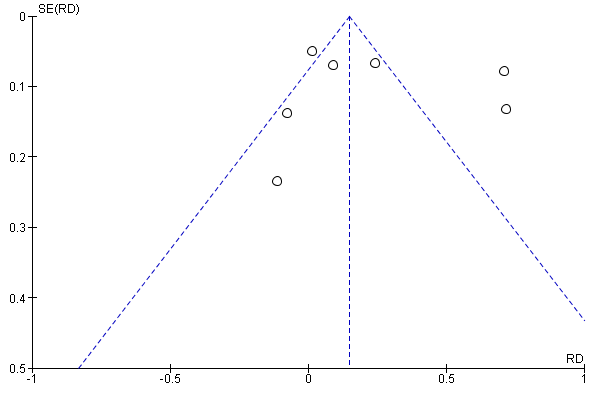


**Fig. S2** Funnel plot of the effects of weight loss on mortality in hospitalized PLHIV

Supplement: Supplementary file 4 — Fig. S2: Funnel plot of the effects of weight loss on mortality in hospitalized PLHIV [file 12879_2023_8889_MOESM4_ESM.docx]

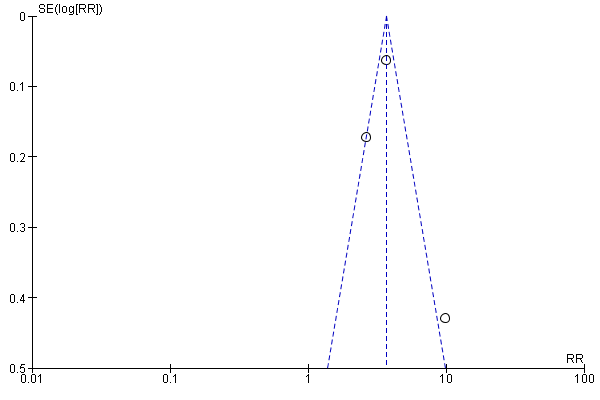


**Fig. S3** Funnel plot of the effects of weight loss on mortality in non-hospitalized PLHIV

Supplement: Supplementary file 5 — Fig. S3: Funnel plot of the effects of weight loss on mortality in non-hospitalized PLHIV [file 12879_2023_8889_MOESM5_ESM.docx]
