# Supplementary material for: Weight loss and mortality in people living with HIV: a systematic review and meta-analysis
Source: BMC Infect Dis. 2024 Jan 2;24:34. doi: 10.1186/s12879-023-08889-3 (PMC10762994; doi:10.1186/s12879-023-08889-3)
Supplement: Supplementary file 6 — Fig. S4: Baujat plot of primary outcome studies [file 12879_2023_8889_MOESM6_ESM.docx]

**
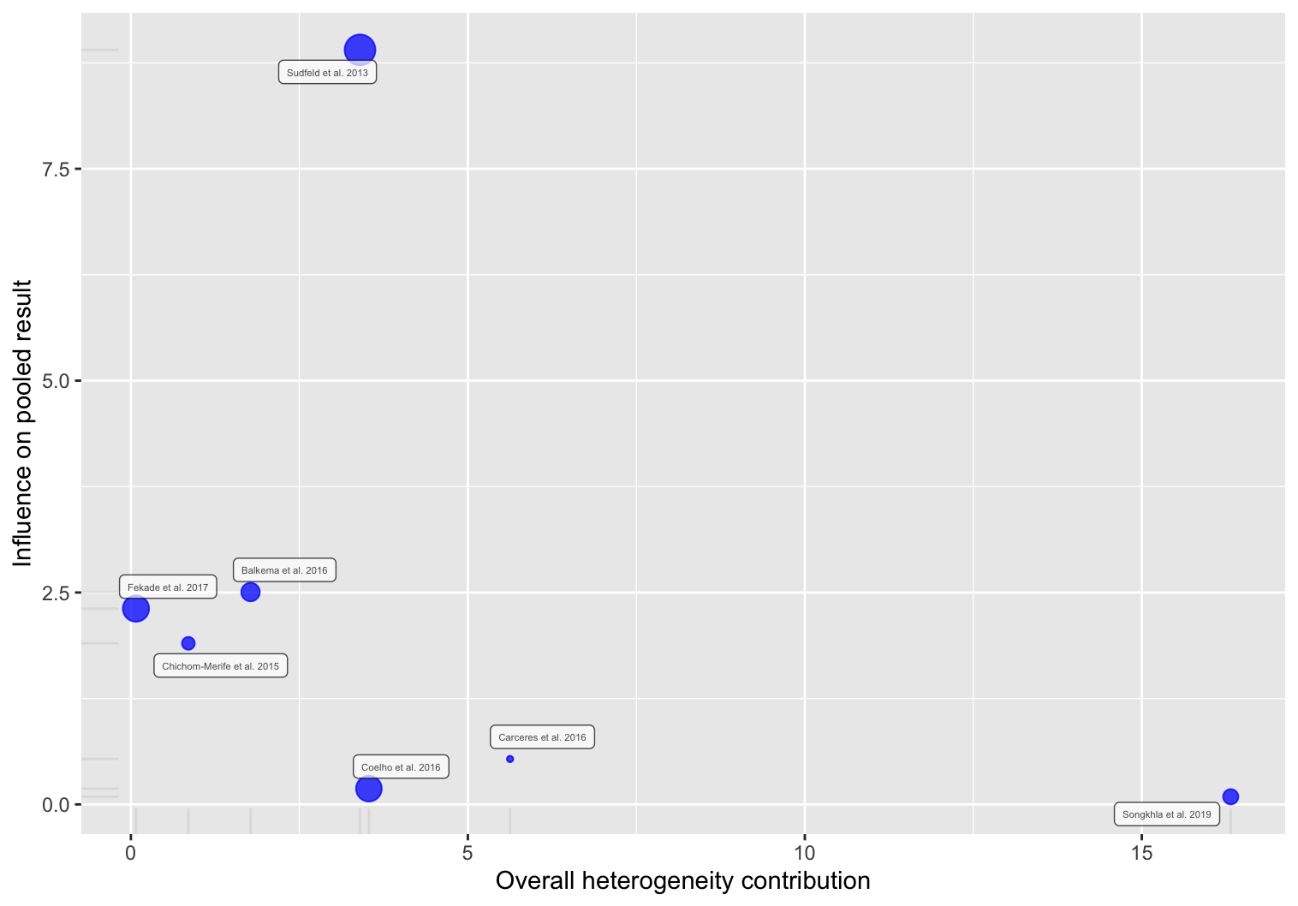
**

**Fig. S4 -** Baujat plot of primary outcome studies

The Baujat plot shows the contribution of each study to the general heterogeneity, on the horizontal axis, and its influence on the combined effect size, on the vertical axis. In this way, the study by Songkhla et al., 2019 was considered potentially relevant, as it strongly contributes to the overall heterogeneity in our meta-analysis. On the other hand, studies focused on the upper part of the graph can be particularly influential, as it has a great impact on heterogeneity in the combined effect size.
