# Supplementary material for: Weight loss and mortality in people living with HIV: a systematic review and meta-analysis
Source: BMC Infect Dis. 2024 Jan 2;24:34. doi: 10.1186/s12879-023-08889-3 (PMC10762994; doi:10.1186/s12879-023-08889-3)
Supplement: Supplementary file 7 — Fig. S5: Influence diagnosis of primary outcome studies [file 12879_2023_8889_MOESM7_ESM.docx]

**
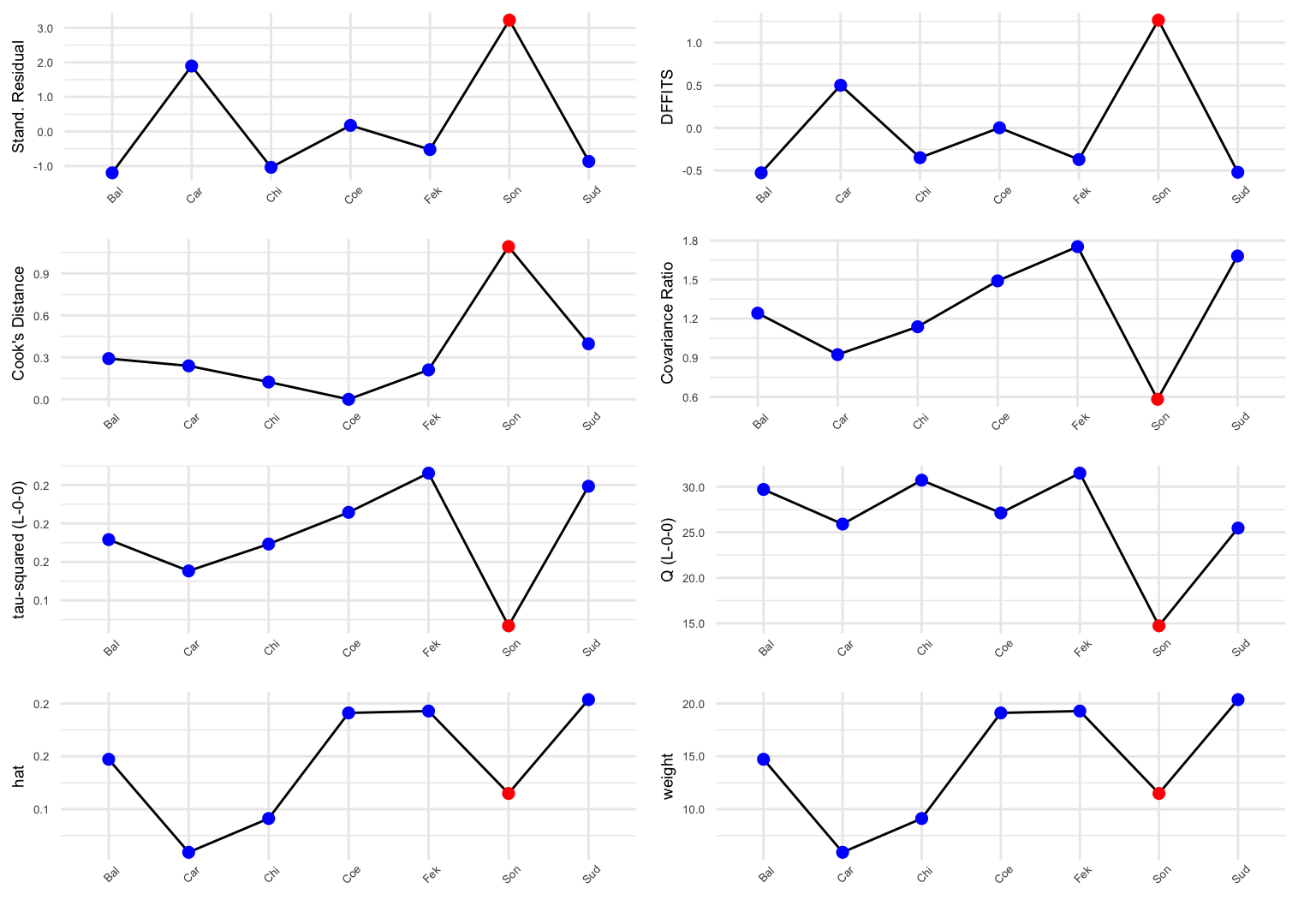
**

**Fig. S5** Influence diagnosis of primary outcome studies

For the diagnosis of influence, the value of different measures was evaluated to characterize which studies fit well in our meta-analysis model and which do not. The measures analyzed were: Externally standardized residues; Calculation DFFITS; Cook's Distance; Covariance Ratio; Tau^2^ (Leave-One-Out); Q Test (Leave-One-Out); Hat value, and study weight. All these measures provide a value that, if extreme, indicates that a study is an influential case and can negatively affect the results. The red circle represents the study by Songkhla et al, 2019 and may show its influence.
