# Supplementary material for: Weight loss and mortality in people living with HIV: a systematic review and meta-analysis
Source: BMC Infect Dis. 2024 Jan 2;24:34. doi: 10.1186/s12879-023-08889-3 (PMC10762994; doi:10.1186/s12879-023-08889-3)
Supplement: Supplementary file 8 — Table S1: Summary of the statistical method, effect estimate and confidence interval by outcome [file 12879_2023_8889_MOESM8_ESM.docx]

**Table S1.** Summary of the statistical method, effect estimate and confidence interval by outcome

| Outcome | Estudies | Participants | Statistical method | Effect estimate |
| --- | --- | --- | --- | --- |
| Primary outcome: Mortality in PLHIV with weight loss Hospitalized | 7 | 489 | Risk Ratio  (M-H, Random, 95% CI) | 1.50 (1.03, 2.19) |
| Secondary outcome: Mortality in PLHIV with weight loss Not hospitalized | 3 | 1148 | Risk Ratio  (M-H, Random, 95% CI) | 3.84 (2.48, 5.95) |

Caption: CI: Confidence interval; M-H: Mantel-Haenszel; RR: Risk ratio
