# Supplementary material for: Weight loss and mortality in people living with HIV: a systematic review and meta-analysis
Source: BMC Infect Dis. 2024 Jan 2;24:34. doi: 10.1186/s12879-023-08889-3 (PMC10762994; doi:10.1186/s12879-023-08889-3)
Supplement: Supplementary file 9 — Table S2: Data for the sensitivity analysis of the primary outcome [file 12879_2023_8889_MOESM9_ESM.docx]

**Table S2.** Data for the sensitivity analysis of the primary outcome

| **Model** | **RR** | **IC 95%** | **p-value** | | ***I*^2^** | **IC 95% (*I*^2^)** | **tau** | **tau^2^** | **H** | **IC 95% (H)** | ***Q* (Chi^2^)** | **d.f** | | ***p*-value** |
| --- | --- | --- | --- | --- | --- | --- | --- | --- | --- | --- | --- | --- | --- | --- |
| Random effects model | 1.50 | 1.03 -2.19 | | 0.04 | 82% | 63.7-90.9 | 0.41 | 0.18 | 2.35 | 1.66 -3.32 | 33.10 | 6 | <0.0001 | |

The variation in heterogeneity between studies was estimated by DerSimonian-Laird; and Q-test (Chi2). RR= risk ratio; CI= confidence interval
