# Supplementary material for: Weight loss and mortality in people living with HIV: a systematic review and meta-analysis
Source: BMC Infect Dis. 2024 Jan 2;24:34. doi: 10.1186/s12879-023-08889-3 (PMC10762994; doi:10.1186/s12879-023-08889-3)
Supplement: Supplementary file 10 — Table S3: Effect of the gender variable on mortality in PLHIV [file 12879_2023_8889_MOESM10_ESM.docx]

**Table S3.** Effect of the gender variable on mortality in PLHIV

| **Predictor** | **Point Estimate** | **Standard Error** | **IC 95% Lower** | **IC 95% Upper** | ***Z*-value** | ***p*-value** |
| --- | --- | --- | --- | --- | --- | --- |
| Intercept* | 0.5494 | 0.3866 | -0.2083 | 1.3071 | 1.4211 | 0.1553 |
| Gender | -0.0036 | 0.0090 | -0.0213 | 0.0140 | -0.4058 | 0.6849 |

* Represents reference level. Mixed-Effects Model (n=7 studies; tau^2^ estimated by: DerSimonian-Laird)
