# Supplementary material for: Weight loss and mortality in people living with HIV: a systematic review and meta-analysis
Source: BMC Infect Dis. 2024 Jan 2;24:34. doi: 10.1186/s12879-023-08889-3 (PMC10762994; doi:10.1186/s12879-023-08889-3)
Supplement: Supplementary file 11 — Table S4: Data from the sensitivity analysis by meta regression [file 12879_2023_8889_MOESM11_ESM.docx]

**Table S4.** Data from the sensitivity analysis by meta regression

| **Model** | **tau** | **tau^2^** | **SE** | ***I*^2^** | **H^2^** | **R^2^** | **QQ** | **d.f** | ***p*-value** | ***Q*M** | **d.f** | ***p*-value** |
| --- | --- | --- | --- | --- | --- | --- | --- | --- | --- | --- | --- | --- |
| Mixed-Effects Model | 0.4595 | 0.2111 | 0.2089 | 84.09% | 6.29 | 0.00% | 31.43 | 5 | <0.0001 | 0.1646 | 1 | 0.6849 |

Source: the authors, 2023
