# Supplementary material for: Weight loss and mortality in people living with HIV: a systematic review and meta-analysis
Source: BMC Infect Dis. 2024 Jan 2;24:34. doi: 10.1186/s12879-023-08889-3 (PMC10762994; doi:10.1186/s12879-023-08889-3)
Supplement: Supplementary file 12 — Table S5: Summary of statistical method, effect estimate and confidence interval by subgroups [file 12879_2023_8889_MOESM12_ESM.docx]

**Table S5.** Summary of statistical method, effect estimate and confidence interval by subgroups

1. Subgroup: Mortality in PLHIV with weight loss by geographic location

| Subgroup | Studies | Participants | Statistical Method | Estimated Effect |
| --- | --- | --- | --- | --- |
| America | 3 | 1110 | Risk Ratio  M-H, Random, 95% CI | 2.90 (1.43, 5.88) |
| Asia | 2 | 142 | Risk Ratio  M-H, Random, 95% CI | 3.64 (1.64, 8.04) |
| Africa | 5 | 385 | Risk Ratio  M-H, Random, 95% CI | - 1. (0.83, 2.41) |

CI: Confidence interval; M-H: Mantel-Haenszel; RR: Risk ratio

1. Subgroup: Mortality in PLHIV with weight loss by study quality

| Subgroup | Studies | Participants | Statistical Method | Estimated Effect |
| --- | --- | --- | --- | --- |
| Moderate risk of bias | 2 | 1034 | Risk Ratio  M-H, Random, 95% CI | 3.97 (2.84, 5.54) |
| Low risk of bias | 8 | 603 | Risk Ratio  M-H, Random, 95% CI | - 1. (1.15, 2.63) |

CI: Confidence interval; M-H: Mantel-Haenszel; RR: Risk ratio

1. Subgroup: Mortality in PLHIV with weight loss by sample size

| Subgroup | Studies | Participants | Statistical Method | Estimated Effect |
| --- | --- | --- | --- | --- |
| N. of sample <100 | 5 | 144 | Risk Ratio  M-H, Random, 95% CI | 2.91 (0.87, 9.69) |
| N. of sample >100 | 5 | 1493 | Risk Ratio  M-H, Random, 95% CI | 1.81 (0.98, 3.34) |

CI: Confidence interval; M-H: Mantel-Haenszel; RR: Risk ratio
