# Supplementary material for: Weight loss and mortality in people living with HIV: a systematic review and meta-analysis
Source: BMC Infect Dis. 2024 Jan 2;24:34. doi: 10.1186/s12879-023-08889-3 (PMC10762994; doi:10.1186/s12879-023-08889-3)
Supplement: Supplementary file 13 — Table S6: Methodological quality and risk of bias assessment [file 12879_2023_8889_MOESM13_ESM.docx]

**Table S6.** Methodological quality and risk of bias assessment

| Author(s), year | Q01 | Q02 | Q03 | Q04 | Q05 | Q06 | Q07 | Q08 | Q09 | Q10 | Q11 | Total “Yes”  N. (%) | Risk of bias |
| --- | --- | --- | --- | --- | --- | --- | --- | --- | --- | --- | --- | --- | --- |
| *Balkema CA et al, 2016* |  |  |  |  |  |  |  |  |  |  |  | 9 (81,8) | Low |
| *Patterson S et al, 2015* |  |  |  |  |  |  |  |  |  |  |  | 7 (63,6) | Moderate |
| *Zhao Y et al, 2017* |  |  |  |  |  |  |  |  |  |  |  | 10 (90,9) | Low |
| *Coelho L et al, 2016* |  |  |  |  |  |  |  |  |  |  |  | 11 (100) | Low |
| *Chichom MA et al, 2015* |  |  |  |  |  |  |  |  |  |  |  | 9 (81,8) | Low |
| *Fekade D et al, 2017* |  |  |  |  |  |  |  |  |  |  |  | 11 (100) | Low |
| *Songkhla MN et al, 2019* |  |  |  |  |  |  |  |  |  |  |  | 7 (63,6) | Moderate |
| *Caceres DH et al, 2016* |  |  |  |  |  |  |  |  |  |  |  | 9 (81,8) | Low |
| *Sudfeld CR et al, 2013* |  |  |  |  |  |  |  |  |  |  |  | 9 (81,8) | Low |
| *Mugusi SF et al, 2012* |  |  |  |  |  |  |  |  |  |  |  | 9 (81,8) | Low |

Caption: Yes No Unclear

Q 01- Were the two groups similar and recruited from the same population? Q 02- Were the exposures measured similarly to assign people to exposed and unexposed groups? Q 03- Was exposure measured in a valid and reliable way? Q 04- Were confounding factors identified? Q 05- Were strategies to deal with confounding factors established? Q 06- Were the groups / participants free of the endpoint at the start of the study (or at the time of exposure)? Q 07- Were the outcomes measured in a valid and reliable way? Q 08- Was the follow-up time reported and sufficient to be long enough for outcomes to occur? Q 09- Was follow-up complete, and if not, were the reasons for loss to follow-up described and explored? Q 10- Were strategies used to deal with incomplete follow-up? Q 11- Was appropriate statistical analysis used?
