# Supplementary material for: Weight loss and mortality in people living with HIV: a systematic review and meta-analysis
Source: BMC Infect Dis. 2024 Jan 2;24:34. doi: 10.1186/s12879-023-08889-3 (PMC10762994; doi:10.1186/s12879-023-08889-3)
Supplement: Supplementary file 14 — Table S7: Evidence profile and summary of results from the GRADE Working Group [file 12879_2023_8889_MOESM14_ESM.docx]

**Table S7.** Evidence profile and summary of results from the GRADE Working Group

1. Primary outcome: Mortality in PLHIV with weight loss Hospitalized

| **Evidence Profile** | | | | | | | **Summary of Results** | | | | |
| --- | --- | --- | --- | --- | --- | --- | --- | --- | --- | --- | --- |
| **Participants**  **(studies)** | **Risk of bias** | **Inconsistency** | **Indirect evidence** | **Imprecision** | **Publication bias** | **Overall Certainty of Evidence** | **Study event rates (%)** | | **Relative Effect**  **(95% CI)** | **Potential absolute effects** | |
|  |  |  |  |  |  |  | **With Comparator: No weight loss** | **With Exposure: Weight Loss** |  | **Risk with Comparator: No weight loss** | **Risk difference with Exposure: Weight loss** |
|  | | | | | | | | | | | |
| 489  (7 observational studies) | Not Severe | Severe^a^ | Not Severe | Severe ^b,c^ | Highly suspect publication bias ^d^ | ⨁◯◯◯ VERY LOW | 208/489 (42.5%) | 281/489 (57.5%) | RR 1.50 (1.03 to 2.19) | 43 per 100 | 21 more per 100  (from 1 more to 51 more) |

Caption: CI: Confidence interval; RR: Risk ratio.

1. Secondary outcome: Mortality in PLHIV with weight loss Not hospitalized

| **Evidence Profile** | | | | | | | | **Summary of Results** | | | | | | | |
| --- | --- | --- | --- | --- | --- | --- | --- | --- | --- | --- | --- | --- | --- | --- | --- |
| **Participants**  **(studies)** | **Risk of bias** | **Inconsistency** | **Indirect evidence** | **Imprecision** | **Publication bias** | **Overall Certainty of Evidence** | | **Study event rates (%)** | | | | **Relative Effect**  **(95% CI)** | | **Potential absolute effects** | |
|  |  |  |  |  |  |  |  | **With Comparator: No weight loss** | | **With Exposure: Weight Loss** | |  |  | **Risk with Comparator: No weight loss** | **Risk difference with Exposure: Weight loss** |
| 1148 (3 observational studies) | Not Severe | Severe ^a^ | Not Severe | Severe ^e,f,g^ | Highly suspect publication bias High association ^d^ | ⨁◯◯◯ VERY LOW | 245/1148 (21.3%) | | 903/1148 (78.7%) | | RR 3.84 (2.48 to 5.95) | | 21 per 100 | | 61 more per 100  (From 32 more to 100 more) |

Caption: CI: Confidence interval; RR: Risk ratio

^a^ There is high heterogeneity between the studies included in the analysis of mortality in hospitalized and non-hospitalized patients (I^2^=82% and I^2^= 78%), respectively. In general, it was decided to downgrade one level when considering this issue. ^b^ One study did not present confounding factors, as well as strategies to deal with them were not established. ^c^ Five studies did not mention the reasons for loss to follow-up and did not report the strategies to deal with incomplete follow-up of their participants. ^d^The population of PLHIV is limited to the studies found from the Pubmed, Embase and LILACS search, and therefore not corresponding to a generalizable sample, making it non-comprehensive, which may have implied publication bias. ^e^In two studies the measurement of the validity and reliability of the exposure was found to be unclear. ^f^ One study did not present the strategies for dealing with confounding factors. ^g^ One study did not mention the reasons for loss to follow-up, as well as the strategies for dealing with incomplete follow-up of its participants. Source: GRADE Working Group - <https://gradepro.org>
